# Chitosan functionalized selenium nanoconjugates of *Mucuna pruriens* as a nanotherapeutic approach for Parkinson’s disease

**DOI:** 10.1186/s11671-026-05114-3

**Published:** 2026-11-04

**Authors:** Saswati Swagatika Sahoo, Srijita Modak, Pratap Kumar Sahu, Anindita Behera, Ketan M. Ranch, Ranjit Prasad Swain

**Affiliations:** 1 School of Pharmaceutical Sciences, Siksha ‘O’ Anusandhan Deemed to be University, Bhubaneswar, Odisha India; 2 Amity Institute of Pharmacy, Amity University, Kolkata, West Bengal India; 3 L. M. College of Pharmacy, Ahmedabad, Gujarat India; 4 GITAM School of Pharmacy, GITAM Deemed to be University, Visakhapatnam, Telangana India

**Keywords:** Mucuna pruriens, Microwave-assisted extraction, Levodopa, Chitosan-selenium nanoparticles, Parkinson’s disease

## Abstract

Nature’s mother holds many untapped treasures of phytoconstituents that can cure and exhibit unexpected therapeutic activity against several diseases. Seeds of velvet beans, popularly known as *Mucuna pruriens* (MP), *are* the richest source of Levodopa (L-DOPA). L-DOPA is the precursor for dopamine, a key neurotransmitter deficient in the brain of patients suffering from Parkinson’s disease (PD). The peripheral degradation of L-DOPA before crossing the blood-brain barrier (BBB) is the greatest challenge in the treatment of PD patients. Several preclinical and clinical studies reported the excellent efficacy of MP seed extracts. To deliver the key constituents of MP seed extracts, the research work designed a nanocomposite containing a trace element (selenium), a natural polysaccharide polymer (chitosan), and MP seed extract. The efficacy of the Chitosan@selenium *Mucuna pruriens* NPs (CSMNPs) was evaluated at two dose levels (25 and 50 mg•kg^− 1^) in the reserpine-induced PD rat model, compared with MP extract and selenium NPs of *Mucuna pruriens* (MSNPs). The surface-modified polymeric metal NPs showcased improved motor function, grip strength, and antioxidant levels in the reserpine-induced PD rat model, significantly. In comparison to MP extract and MSNPs, the CSMNPs showed better antiparkinsonian and antioxidant properties, which may be attributed to their smaller size and better permeability through the BBB. Histopathological assessment of the hippocampus and the substantia nigra of the brain also supported the results of CSMNPs.

## Introduction

Parkinson’s disease (PD) is the second most common neurodegenerative disorder after Alzheimer’s disease and is primarily characterized by progressive motor dysfunction, including tremor, rigidity, bradykinesia, and postural instability [1, 2]. The pathophysiology of PD is mainly associated with the progressive degeneration of dopaminergic neurons in the substantia nigra pars compacta, resulting in dopamine depletion within the striatum [3, 4]. Since dopamine plays a central role in the regulation of basal ganglia circuits, its depletion disrupts motor control and contributes to the characteristic motor abnormalities observed in PD. In addition, accumulation and aggregation of misfolded α-synuclein protein in the form of Lewy bodies is considered a major histopathological hallmark of PD [5, 6]. Several interrelated mechanisms, including mitochondrial dysfunction, oxidative stress, neuroinflammation, excitotoxicity, and impaired protein clearance, further contribute to neuronal injury and disease progression [7]. PD may also involve non-motor manifestations, including cognitive impairment, sleep disturbances, mood alterations, autonomic dysfunction, and olfactory abnormalities. The occurrence of PD varies worldwide and increases with age. According to WHO data in 2019, globally, over 8.5 million individuals were affected by PD. Due to ageing populations worldwide, the prevalence of PD is expected to double by 2030, making it an increasingly significant public health concern (WHO) [8]. Currently, pharmacological management of PD mainly focuses on symptomatic relief. Levodopa (L-DOPA) remains the gold-standard therapy and is commonly administered either alone or in combination with carbidopa to enhance central dopamine availability [9]. Long-term use of levodopa is also associated with complications such as dyskinesia, motor fluctuations, hallucinations, and reduced therapeutic efficacy over time [10]. Additional therapies such as dopamine agonists, monoamine oxidase-B (MAO-B) inhibitors, COMT inhibitors, and anticholinergic drugs may temporarily improve motor symptoms, but they do not halt the disease progression, highlighting the need for safer and more effective neuroprotective strategies for PD management.

*Mucuna pruriens* (L.) DC. (MP), commonly known as velvet bean, is a tropical climbing leguminous plant belonging to the Fabaceae family and is an annual plant native to India [11]. It contains several bioactive compounds, including serotonin, mucunain, and proteins. The seeds of *M. pruriens* are a natural source of L-DOPA, a potent neurotransmitter and an uncommon non-protein amino acid [12]. The seeds of MP have traditionally been used in the management of PD due to their dopamine-enhancing properties [13]. MP seeds are reported to exhibit anticataleptic [14] and antiparkinsonian activities, typically containing 4–6% L-DOPA [15–17]. In vitro studies demonstrate that MP seeds significantly enhance the activity of brain mitochondrial complex I without affecting the activity of total monoamine oxidase. This is attributed to the presence of coenzyme Q-10 and nicotinamide adenine dinucleotide (NADH) [18]. Furthermore, MP seed powder has been found to restore endogenous levels of L-DOPA, dopamine, norepinephrine, and serotonin in the substantia nigra more effectively than synthetic L-DOPA treatment [19]. However, there are currently no standardised procedures to ensure the purity and potency of MP extracts.

Nanoparticles (NPs) are the most efficient and potent drug delivery systems for the brain [20, 21]. They deliver therapeutic agents with more efficient targeting to deliver them into the damaged areas. Selenium (Se) is an essential trace element that plays a fundamental function in numerous physiological processes. It is a key element of selenoproteins, such as glutathione peroxidases (GPx), which safeguard cells from damage caused by oxidation by converting lipids and hydrogen peroxide (H_2_O_2_) into safe compounds [22, 23]. Se NPs may reduce oxidative damage in the brain by inducing calcium signalling pathways associated with PD [24]. Se NPs may reduce oxidative damage in the brain by inducing calcium signalling pathways associated with PD. Because of their antioxidant qualities, Se NPs are being investigated for possible application in PD [7, 25–28].

In particular, polymeric NPs can be utilised as brain-targeted drug delivery systems to facilitate the controlled release and delivery of both hydrophilic and hydrophobic drugs [29–31]. Chitosan (CS) is a natural polysaccharide that has been used mostly in the synthesis of NPs [32–35]. CS has received increasing attention for synthesizing polymeric NPs because of its mucoadhesive properties, low toxicity, biocompatibility, and biodegradability [36–38]. It biodegrades more quickly when its degree of deacetylation is less than 70% [39]. CS and its surface modifications on NPs offer numerous benefits, including improving drug absorption and controlled drug release. Additionally, CS coating reduces the burst release of the drug. The interaction between the anionic plasma membrane and cationic CS causes NPs to permeate and be retained more [40–43]. Low-molecular-weight CS has better aqueous solubility than high-molecular-weight CS. The encapsulation efficiency of CS increases with a decrease in the molecular weight [44].

MP seeds are established to exhibit antiparkinsonian activity [45–47]. The poor stability, bioavailability, and limited permeation through the blood-brain barrier (BBB) restrict the clinical application of M. pruriens in the management of PD. Metal and polymeric NPs have been synthesized and studied to overcome these limitations, enhancing their efficacy and safety [15, 46–51]. Hence, we hypothesized that CS-coated Se NPs of MP seed extract may enhance the antiparkinsonian efficacy of MP seed extract in rats by improving drug delivery across the BBB, reducing oxidative stress, and promoting neuronal survival.

This study was designed to prepare polymer-metal NPs containing chitosan and selenium-conjugated *Mucuna pruriens* nanoparticles (CSMNPs) and study their efficacy in reserpine-induced PD in a rat model. The Se nanoparticles of the aqueous seed extract of MP (MSNPs) were prepared using a chemical reduction technique, followed by the conjugation of metal NPs to CS to enhance stability, bioavailability, and permeability through the BBB. The study also included the evaluation of the antioxidant efficacy of CSMNPs using in vivo and in vitro methods, as well as biochemical estimations of AChE and nitrite levels. Additionally, the improvement of PD symptoms was assessed through neurobehavioral evaluation in reserpine-induced PD rat models.

## Experimental methods

### Materials, chemicals, and reagents

MP seeds were procured from Raj and Company, Neemuch (Madhya Pradesh), bearing the voucher specimen number 2115/CBT. Chemical reagents like sodium selenite and CS (catalogue no. GRM9358, CAS No. – 9012-76-4, from shrimp shells, molecular weight 3800–20000 Daltons, degree of deacetylation ≥ 75%) were acquired from Sigma-Aldrich, India, and Himedia Laboratories Pvt. Ltd., India, respectively. The CS we have used for the experiments is low-molecular-weight CS (3800 to 500,000 Daltons). The viscosity for the 1% w/v solution in acetic acid (25% v/v) at 500 °C was found to be 172 cps. The certificate of analysis we received for low molecular weight CS from Sigma-Aldrich indicates a viscosity of 172 cps, which is similar to the experimental values; therefore, the CS was used as received without any purification. The molecular weight of CS is typically expressed as a range rather than an exact value. This is because the degree of acetylation of CS significantly influences its molecular weight [52]. Ascorbic acid, L-DOPA, and reserpine were procured from Sigma-Aldrich, India. All the chemicals and solvents required for the in vitro and in vivo experimental work were purchased from authentic dealers of Merck, India.

### Synthesis of *Mucuna pruriens*-selenium NPs (MSNPs) and Chitosan@selenium *Mucuna pruriens* NPs (CSMNPs)

#### Preparation of aqueous seed extract of MP

MP seeds are procured, cleaned, dried, and wiped to remove dust particles from their surface. The seeds were air-dried for 48 h. Then, 500 mg of fine seed powder was taken in a 250 mL beaker, soaked in a mixture of 50 mL of HPLC-grade water, 0.5 mL of glacial acetic acid, and 0.05 g of sodium sulfide. An aqueous extract was prepared using a home microwave powered at 800 watts, in three cycles of one minute each, separated by a one-hour interval. The extract was filtered, collected and stored under refrigeration for later use [53].

#### Qualitative and quantitative evaluation of L-DOPA in the seed extract of *MP*

The extract was diluted in the ratio 1:10000 for both qualitative analysis and quantitative estimation of L-DOPA in the seed extract of MP. Standard L-DOPA was used for the qualitative and quantitative estimation of L-DOPA in the MP seed extract by UV-visible spectrophotometry. A primary standard L-DOPA solution (100 µg/mL) was used to prepare serial dilutions ranging from 5 to 25 µg/mL. A test solution was also made by diluting 0.01 mL of the extract to 100 mL. The λ_max_ of the standard and test solutions was detected spectrophotometrically (JASCO, V-630). An overlay of the spectrum can identify and confirm the L-DOPA content in MP seed extract. The L-DOPA content in the seed extract was calculated using the standard calibration curve and reported as a percentage (w/w).

#### Preparation of selenium NPs of *Mucuna pruriens* (MSNPs)

Selenium NPs conjugated with the seed extract of MP were prepared using the green synthesis method, which does not involve any use of harmful chemical reagents. The seed extract functioned as both a reductant and stabiliser in the synthesis of MSNPs. Sodium selenite pentahydrate (Na₂SeO₃·5 H₂O, 0.5 mM) solution served as the metal precursor and was combined with the diluted seed extract (B) in a 9:25 ratio. The reaction was performed by maintaining the temperature at 50–60 °C and continuously stirring at a speed of 500–600 rpm until a noticeable colour change occurred. The transition from colourless to orange indicated the formation of the NPs, and the time required for this change was recorded.

#### Preparation of Chitosan@selenium *Mucuna pruriens* NPs (CSMNPs)

An acetic acid (1% v/v) solution was added to 10 mL of HPLC-grade water to dissolve 0.01 g of CS at room temperature. The CS was dissolved for 1 h at 60–70 °C. Then, the CS solution was added to MSNPs at 40–50 °C with constant stirring for 2 h until the colour of the reaction medium transformed from orange to light pink.

### Characterization of MSNPs and CSMNPs

The green synthesis method was employed to produce MSNPs and CSMNPs. Ascorbic acid was used to stabilise and reduce the polymer-conjugated selenium nanoparticles (Se NPs). The reaction medium temperature, stirring duration and speed, sequence of addition, and volumes and concentrations of reagents and sample solutions were meticulously optimised. Each parameter was validated in triplicate to ensure consistent and reproducible results. The formation of MSNPs and CSMNPs was confirmed through surface plasmon resonance (SPR) analysis using a UV-visible spectrophotometer (JASCO, V-630). Additional characterisation of the samples was accomplished using Fourier transform infrared spectroscopy (FTIR), measurements of hydrodynamic diameter, surface charge, and stability, X-ray diffraction (XRD), and high-resolution transmission electron microscopy (HRTEM) coupled with selected area electron diffraction (SAED) imaging.

The solutions of synthesised NPs were scanned from 200 to 400 nm to identify the SPR bands to evaluate the optical properties of MSNPs and CSMNPs compared to the aqueous extract [7]. The aqueous extract, MSNPs, and CSMNPs were analysed using the ATR method with an FTIR instrument (JASCO FTIR- 4600). The samples were directly mounted on an ATR crystal and scanned in the mid-infrared region of 4000 to 400 cm^− 1^ to detect the functional groups and assess the chemical interactions [18]. The hydrodynamic diameter and polydispersity index of MSNPs and CSMNPs were evaluated using a Zeta sizer (Nano-ZS, Malvern) by dispersing the NPs in a suitable medium [54]. The same instrument was used for evaluation of surface charge and colloidal stability of the synthesized NPs in an electrophoretic light scattering mode. The liquid NPs sample was pipetted out, thoroughly agitated, and then dropped onto a 1 cm x 1 cm glass substrate for XRD investigation. A thick layer of sample formed because the procedure was carried out repeatedly, five times, after air drying. After that, the substrate was placed on an amorphous sample holder and firmly fixed on a goniometer’s sample stage. An X-ray diffractometer (Bruker Model D8 Advance A25), equipped with Cu Kα radiation (λ = 1.5406 Å), with the current and voltage set to 40 mV and 40 mA, respectively, was used for data collection [55]. The diffraction pattern was recorded over a 2θ range of 10–80°, with a step size of 0.02° and a scanning rate of 1–2° min⁻¹. The characteristic diffraction peaks were analysed to determine the crystallinity, crystalline phase, and structural characteristics of the nanoparticles. The size, shape, and crystallinity of MSNPs and CSMNPs were confirmed from the HRTEM-SAED images using the JEOL model (JM2100) operated at 200 kV [54]. Samples were dispersed in a suitable organic solvent, sonicated, and deposited onto carbon-coated copper grids. HRTEM images were used to determine particle morphology and size. SAED patterns obtained from selected regions were used to confirm crystallinity and identify the corresponding crystallographic planes.

### Estimation of entrapment efficiency (EE) and drug loading capacity (LC) of L-DOPA in MSNPs and CSMNPs

The MSNPs and CSMNPs were subjected to centrifugation for 30 min at 4 °C and 18,000 rpm. The EE and LC in the NPs were determined using Eqs. (1) and (2), respectively, after the supernatant layer was carefully transferred and examined spectrophotometrically at 280 nm (the absorption maximum of L-DOPA).

1 $${\mathrm{Entrapment}}\,{\mathrm{efficiency}}\,(\%)=[({\mathrm{Td}}-{\mathrm{Fd}})/{\mathrm{Td}}]\times100.$$

2 $${\mathrm{Drug}}\,{\mathrm{loading}}\,{\mathrm{capacity}}\,(\%)\,=\,({\mathrm{Td}}-{\mathrm{Fd}}/{\mathrm{weight}}\,{\mathrm{of}}\,{\mathrm{NPs}})\times100.$$

Where Td = total drug and Fd = free drug.

### In vitro models of free radical scavenging of DPPH and ABTS for antioxidant study

An antioxidant is any compound capable of preventing oxidative damage caused by free radicals. An antioxidant study of MP extract, MSNPs, and CSMNPs was carried out using in vitro models of scavenging free radicals of DPPH and ABTS. The linear regression equation was used to determine the IC_50_ values for the standard, MP extract, MSNPs, and CSMNPs.

#### DPPH scavenging assay

Methanol was used to prepare a standard DPPH solution. The test solutions were made by mixing various dilutions of MP extract. MSNPs and CSMNPs were evaluated for their antioxidant potential in the concentration range of 1 to 30 µg/mL. After an incubation period of 30 min in the dark, the absorbances of standard (ascorbic acid) and test solutions were recorded at 515–517 nm at room temperature [51]. The scavenging activity of the samples was compared to the standard, and the percentage inhibition was calculated using the specified formula.

3 $$\%\,{\mathrm{of}}\;{\mathrm{scavenging}}\,{\mathrm{of}}\,{\mathrm{DPPH}}\,{\mathrm{free}}\,{\mathrm{radicals}}\,(\%)\,=[({\mathrm{A}_{\mathrm{control}}}-{\mathrm{A}_{\mathrm{sample}}})]/({\mathrm{A}_{\mathrm{control}}})]\times100.$$

Where A_control_ = absorbance of methanolic DPPH.

A_sample_ = absorbance of sample or standard in methanolic DPPH.

#### ABTS scavenging assay

The ABTS stock solution (prepared by mixing equal volumes of potassium persulfate (2.45 mM) and ABTS (7 mM)) was incubated in the dark for 12 to 16 h. The ABTS^•+^ stock solution was diluted with distilled water to prepare a working solution, ensuring an absorbance of 0.7 ± 0.005 at 730 nm. To assess the capacity of NPs to scavenge free radicals, diluted ABTS^•+^ solution (1 mL) was mixed with varying amounts of standard (ascorbic acid), MP extract, MSNPs, and CSMNPs, varying from 1 to 30 µg/mL. The final volume of each solution was made up to 5 mL with distilled water. The solutions were incubated for 30 min, and their absorbances were recorded at 730 nm. Then, using the proposed formula, the percentage of ABTS free radical scavenging capacity for the MP extract, MSNPs, and CSMNPs was determined and compared to that of ascorbic acid, as shown in Eq. (3) [56, 57].

### In vivo studies

#### Experimental designs

48 no. of adult Wistar rats of either sex, aged 8–10 weeks, having a weight in the range of 180–200 g, were acquired from the central animal house of the School of Pharmaceutical Sciences, Siksha ‘O’ Anusandhan (Deemed to be University), Bhubaneswar, and properly kept in polypropylene cages. Proper food and water were supplied to them without restrictions. Before the experiment, controlled environmental conditions were maintained, such as a 12-hour light-dark cycle, a temperature of around 25 ± 5 °C, and a relative humidity of 25–35%. Additionally, the animals must have had enough time to adjust to the laboratory environment before the experiments commenced. As stated in the project proposal No. IAEC/SPS/SOA/132/2023, all experimental procedures were carried out in compliance with the authorised protocols and closely followed the guidelines of the IAEC (Institutional Animal Ethics Committee), which has been approved by the CPCSEA. The ARRIVE criteria are compliant with the CPCSEA requirements [58, 59].

Group I - Control (normal saline).

Group II - Negative control treated with reserpine [0.2 mg•kg^− 1^, intraperitoneal (i.p.)]

Group III - Reserpine + L-DOPA (100 mg•kg^− 1^, p.o.)

Group IV - Reserpine + MP seed extract (50 mg•kg^− 1^, p.o.)

Group V - Reserpine *+* MSNPs (25 mg•kg^− 1^, p.o.)

Group VI - Reserpine *+* MSNPs (50 mg•kg^− 1^, p.o.)

Group VII - Reserpine + CSMNPs (25 mg•kg^− 1^, p.o.)

Group VIII - Reserpine + CSMNPs (50 mg•kg^− 1^, p.o.)

**Fig. 1:**
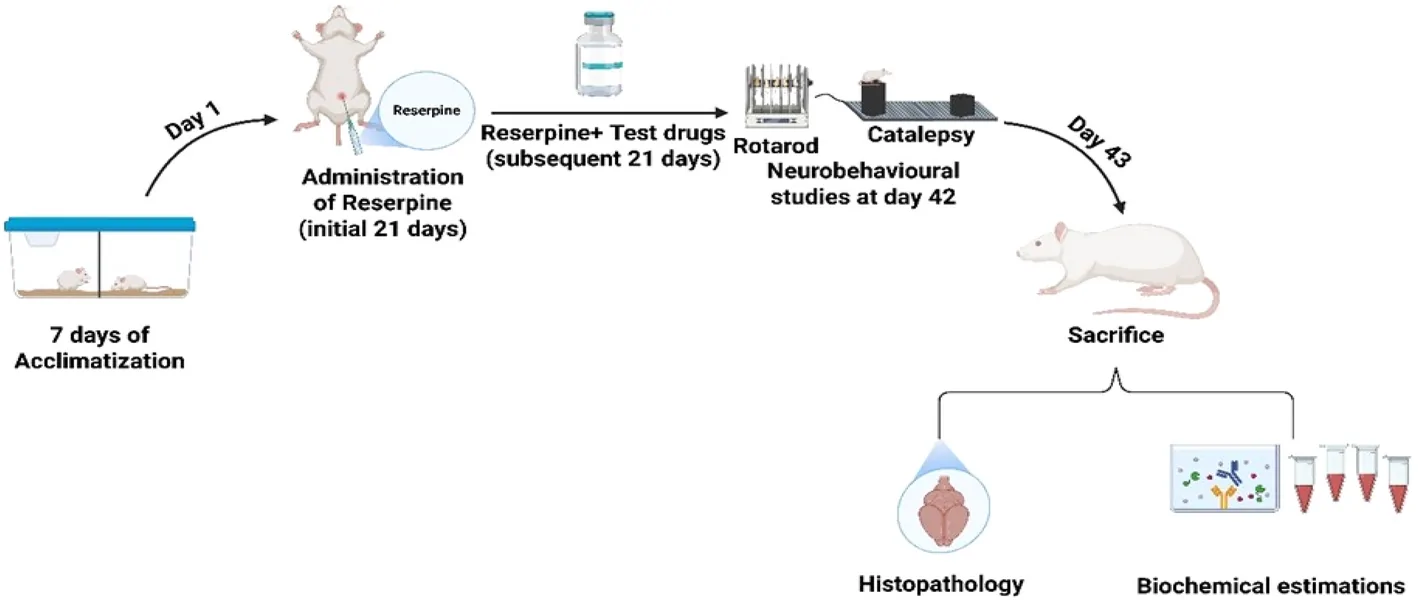
Schematic representation of reserpine-induced experimental protocol, including neurobehavioral assessments, biochemical and histopathological investigations

48 rats were randomly divided into eight experimental groups. Group I was administered normal saline for 42 days and was treated as a control group; Group II was administered reserpine 0.2 mg•kg^− 1^ (i.p.) once a day for 42 days. Groups III-VIII were administered reserpine (0.2 mg•kg^− 1^, i.p.) for the initial 21 days to induce Parkinsonian symptoms. From day 22 to day 42, these groups received reserpine (0.2 mg·kg⁻¹, i.p.) along with their respective test drug, administered at an interval of 1 h between the two administrations. Group III received L-DOPA (100 mg•kg^− 1^, p.o.); Group IV received MP seed extract (50 mg•kg^− 1^, p.o.); Group V and VI received MSNPs at doses of (25 and 50 mg•kg^− 1^, p.o.) respectively; while Group VII and VIII received CSMNPs at doses of (25 and 50 mg•kg^− 1^, p.o.) respectively. The neuropharmacological behavioural assessments were performed on the 42^nd^ day (*n* = 6). On the forty-third day, the animals were sacrificed. Brain homogenate (*n* = 3) was prepared by isolating the whole brain, while the remaining three brains (*n* = 3) were preserved in formalin solution for histopathological analysis. Brain homogenate was used to estimate the in vivo antioxidant status and to perform other biochemical tests (Fig. 1).

#### Neuropharmacological study

Pharmacological investigations, including the rotarod, wooden block, and Y-maze, were used to evaluate different screening models in the PD rat model.

##### Grip strength test

The grip strength test was conducted using a rotarod apparatus to assess motor coordination of the rats [60]. Rats are frequently subjected to the rotarod test to ascertain their “minimal extrapyramidal deficit”, which assesses balance and muscular function. Each rat underwent a training session to become accustomed to the rotarod apparatus before the therapy commenced. The animal was placed on a revolving rod that turned at 20 rpm, & the time it took for the rats to fall from the rotating rod was their time of fall (in seconds), which was recorded [61].

##### Catalepsy test

The catalepsy test was evaluated using a wooden block. Reserpine has a notable cataleptic impact among antipsychotics. Reserpine (0.2 mg•kg^− 1^) was administered by the i.p. route for the induction of catalepsy. For the measurement of catalepsy score, the forelimbs of the rats were placed on a wooden bar of a 1 cm board and 3 to 9 cm high. If the animal fails to regain its posture for ten seconds or more, it is deemed cataleptic. The score would be zero if the rat were left on the table with unrestricted movement. The score is 0.5 if the rat moves when pushed. Place the rat’s front right paw three centimetres up on a wooden platform. The animal would receive 0.5 points for each alternate paw, with a score of 1 if it did not move its paw for at least 10 s. When the rat’s right paw is positioned on the wooden platform, and if it is unable to remove its front paws, the score is 1, and the total score is 2 for each alternating paw [62]. The total score for a Parkinsonian animal should be 3.5 (fully induced PD), or 3.5 points for the severity of the disease. Healthy animals received a score of 0.

#### Brain homogenate preparation method

On the forty-third day, the rats were humanely euthanised using the cocktail solution of (ketamine 87.5 mg/kg and xylazine 12.5 mg/kg i.p.) to induce deep anaesthesia, followed by confirmation of death by cessation of respiration and cervical dislocation, ensuring minimal pain and distress. All procedures were carried out in accordance with Institutional Animal Ethics Committee (IAEC) approval and CPCSEA guidelines. Brain samples from each experimental group (*n* = 3) were collected and preserved in 10% formalin for histopathological examination. Additionally, to produce brain homogenates for antioxidant testing, the remaining brain tissues (*n* = 3) were properly washed with cold saline on ice. After weighing the entire brain, it was homogenised in a 0.3 M phosphate buffer solution (pH 7.4) of a volume equivalent to three times the brain’s weight using an oscillator homogeniser at a frequency of approximately 180–1800 rpm. After that, the resultant homogenates were subjected to centrifugation at 4 °C for 30 min at 10,000 rpm in a cooling centrifuge. The supernatant was subsequently collected and kept at -80 °C for further biochemical estimation [63].

#### In vivo antioxidant studies

##### Estimation of superoxide dismutase (SOD)

Superoxide dismutase (SOD) is a key antioxidant enzyme that facilitates the transformation of superoxide radicals into H_2_O_2_ and oxygen. The SOD level was determined spectrophotometrically at 420 nm. For the blank solution, EDTA solution (0.5 mL, 1 mM), Tris buffer (1.5 mL, 0.05 mM) and pyrogallol (1 mL, 0.2 mM) were mixed, and its absorbance was recorded at 420 nm. The test solution comprised a mixture of the brain homogenate (20 µL) and the blank solution, and the absorbance was recorded at 420 nm. Equation (4) was used to quantify the percentage of protection [64, 65], and the SOD level was determined and expressed as mmol/mg protein [66, 67].

4 $$\%\,{\mathrm{Protection}}\,=\,({\mathrm{A}_{\mathrm{b}}}-{\mathrm{A}_{\mathrm{s}}})/{\mathrm{A}_{\mathrm{b}}}\times100.$$

Where A_b_ = absorbance of blank A_s_ = absorbance of samples.

##### Estimation of MDA (extent of lipid peroxidation)

Malondialdehyde (MDA) is a crucial marker of lipid peroxidation and oxidative stress brought on by free radicals within cells. The blank solution was prepared by mixing hydrochloric acid (HCl) (2 mL, 0.25 N), 15% (v/v) trichloroacetic acid, and 0.37% (w/v) thiobarbituric acid. The test solution comprised a mixture of the brain homogenate (100 µL) and the blank solution, and the absorbance was recorded at 532 nm. Equation (4) was used to determine the MDA concentration, expressed in nmol/mg protein [65].

##### Estimation of catalase (CAT) content

The rate of breakdown of H_2_O_2_ by oxidation was measured to assess catalase (CAT) enzyme activity at 240 nm. The blank solution consisted of a mixture of H_2_O_2_ (1 mL, 0.019 M) and phosphate buffer (pH = 7.4, 1.95 mL, 0.05 M). The test solution comprised a mixture of the brain homogenate (50 µL) and the blank solution, and its absorbance was recorded at 240 nm and compared to the blank. The catalase content was expressed in nmol/mg/protein [54, 68].

##### Estimation of glutathione (GSH) content

The spectrophotometric technique was used to determine the glutathione content. 1 mL of phosphate buffer solution (0.1 M, pH 7.4) was mixed with 1 mL of 5,5’-dithiobis-(2-nitrobenzoic acid) (DTNB) (10 mM) to prepare a blank solution. 100 µL of brain homogenate was shaken continuously with a blank to prepare the test solution having a pale yellow colour. The absorbance was recorded within 15 min at a wavelength of 412 nm and expressed in (µmol/mg protein) [69].

##### Estimation of nitrite level

Using nitrite as an indicator, the Griess reagent was used to measure nitrosative stress in the brain spectrophotometrically at 546 nm. To prepare the test mixture, in a test tube, 500 µL of Griess reagent (a mixture of 0.1% naphthylamine diamine dihydrochloride and 1% sulphanilamide, both diluted in phosphoric acid in a 1:1 ratio) was combined with brain homogenate (100 µL), and the absorbance was recorded. A standard calibration curve of sodium nitrite was used to quantify nitrite ions in the brain [70].

### Histopathological study

For histological analysis, brain samples (*n* = 3) were collected and preserved in a formalin solution (10% v/v). The tissues were subsequently washed, dehydrated using graded ethanol and water solutions, and treated with xylene. The samples were then sectioned into thin slices of 5–6 μm thickness, embedded in paraffin, and stained with eosin and haematoxylin. The dyed tissues were examined using a trinocular biological microscope (MLX, Magnus Olympus, India) at 10x magnification.

### Statistical analysis

The mean ± SD was used to express the above calculations. One-way and Two-way ANOVA was used to analyse the data, and Tukey’s post hoc t-test was then used. The significance threshold (*p* < 0.05) was taken into consideration [71]. Additionally, Cohen’s d value was calculated to determine the practical and biological significance of a therapy by quantifying the size of treatment effects irrespective of sample size. The calculation of Cohen’s d involves dividing the difference between the two group averages by the pooled standard deviation. According to standard criteria, effect sizes were classified as negligible (< 0.20), small (0.20–0.49), medium (0.50–0.79), and high (≥ 0.80).

## Result

### Preparation of MP seed extract

#### Extraction of L-DOPA from seeds of MP by microwave-assisted method

The MP seed extract used for the study was prepared by soaking 500 mg of MP seed powder in 50 mL of HPLC-grade water containing 0.5 mL of glacial acetic acid and 0.05 g of sodium sulfide. The solution was subjected to microwave-assisted extraction, and a clear extract was filtered, collected, and stored for further study.

#### Qualitative and quantitative estimation of L-DOPA in the aqueous seed extract of MP

**Fig. 2:**
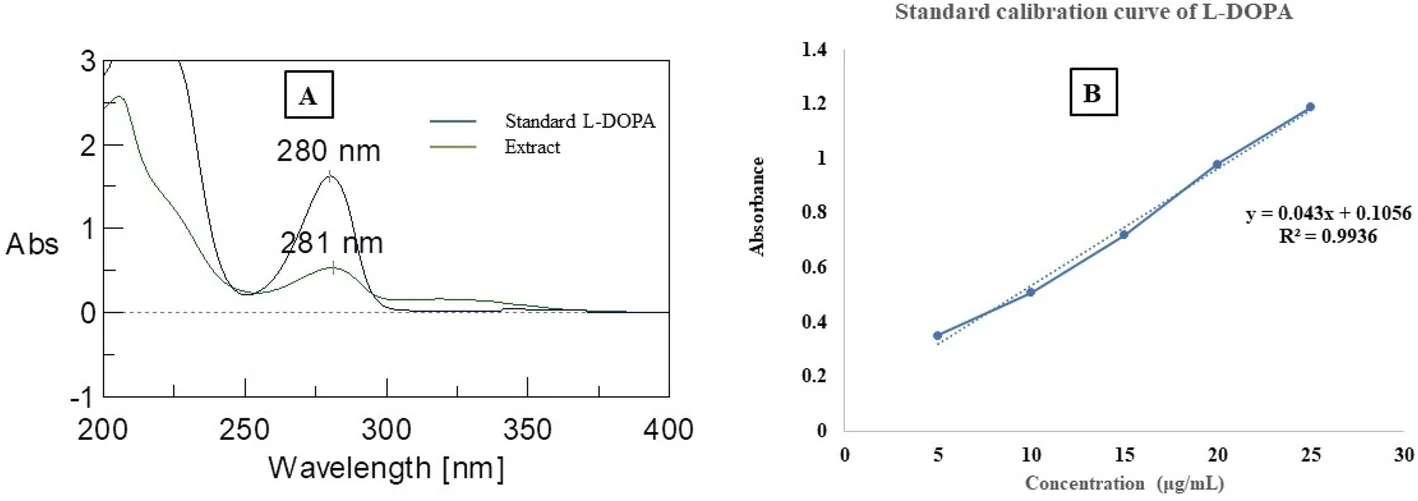
**A** Overlay of standard L-DOPA solution and extract, where the λ_max_ of the standard is 280 nm and λmax of the extract is 281 nm; **B** Standard calibration curve of standard L-DOPA

For qualitative identification of L-DOPA in the seed extract of MP, standard L-DOPA solution and diluted extract were scanned from 200 to 400 nm, and the overlay of the spectrum identified L-DOPA in the extract, as represented in Fig. 2A.

The quantitative estimation of L-DOPA was done from the standard calibration curve plotted as absorbance vs. concentration (µg/mL) of L-DOPA (reference standard) in the 5–25 µg/mL range represented in Fig. 2B. The L-DOPA content in the extract was estimated to be 27.5% w/w.

### Synthesis of MSNPs and CSMNPs

**Fig. 3:**
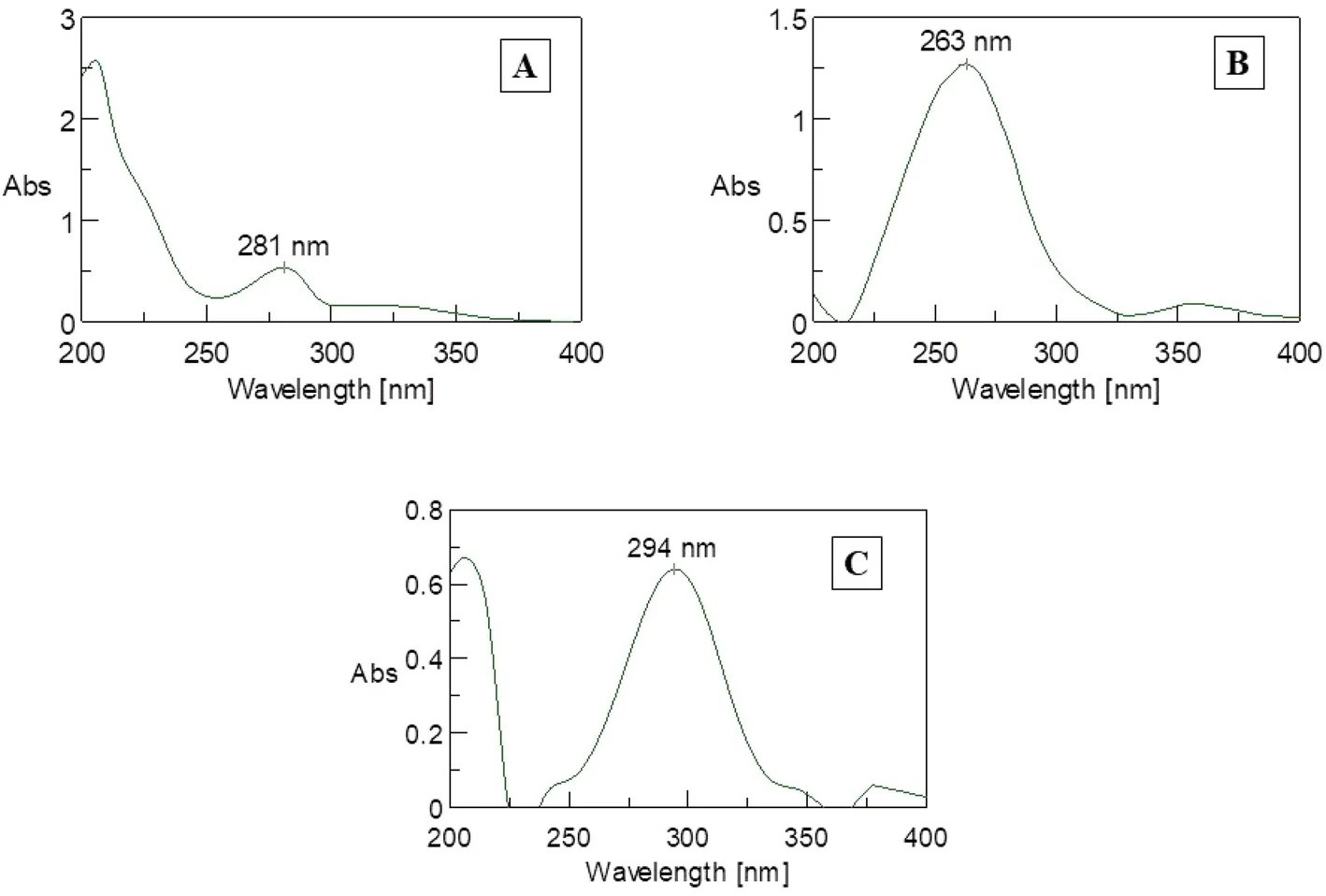
**A** Absorption maxima of MP extract (281 nm); **B** Surface Plasmon Resonance of MSNPs by UV-visible Spectroscopy (263 nm); **C** Surface Plasmon Resonance of CSMNPs by UV-visible Spectroscopy (294 nm)

The synthesis of NPs was confirmed by monitoring the change in colour of the metal precursor solution with seed extract and measuring the SPR band using an ultraviolet-visible spectrophotometer. In the case of MSNPs, adding the extract to the metal solution changed the colour from grey to orange within 30 min. The SPR band of MSNPs was examined by scanning from 200 to 400 nm, and a peak was observed at 263 nm, confirming the synthesis of MSNPs (Fig. 3A). After that, the CS solution (0.1% w/v) was added to MSNPs at 40–50 °C with continuous stirring for 2 h, until the colour changed from orange to light pink. The change of colour from orange to light pink confirmed the synthesis of CSMNPs. UV-visible spectroscopy of CSMNPs showcased the SPR band at 294 nm (Fig. 3B).

### Characterisation of MP extract, MSNPs & CSMNPs

The synthesis of MSNPs and CSMNPs was performed and compared using spectrophotometric methods (UV-visible spectrophotometer and FTIR) to ensure the formation of NPs. The UV-visible spectrophotometric scanning of MP extract, MSNPs, and CSMNPs indicated that significant changes in the λ_max_ confirmed the formation of NPs (Fig. 3). The λ_max_ for MP extract, MSNPs, and CSMNPs were at 281 nm, 263 nm, and 294 nm, respectively (Fig. 3A and B, and 3C).

**Fig. 4:**
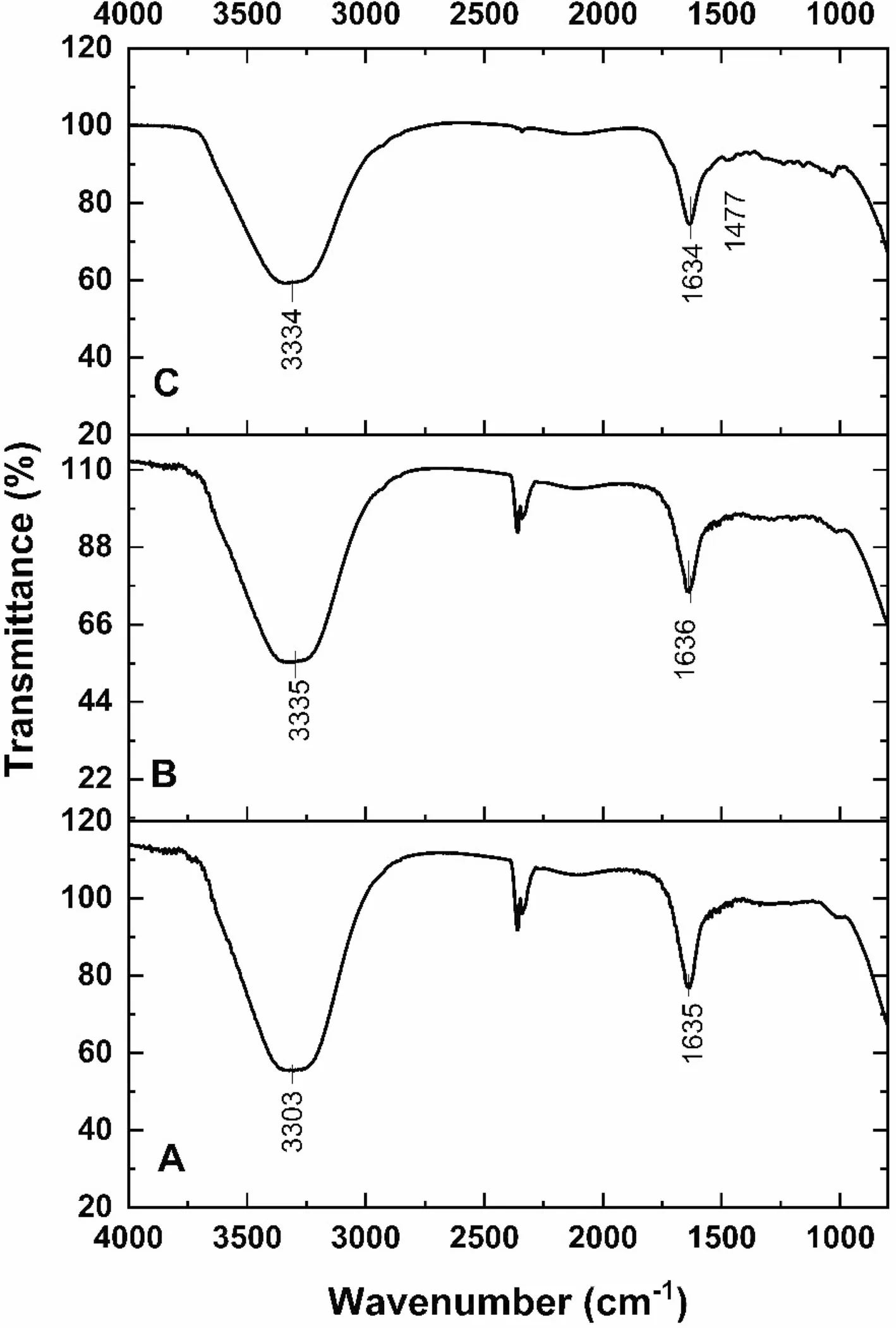
**A** FTIR spectrum of MP extract; **B** FTIR spectrum of MSNPs; **C** FTIR spectrum of CSMNPs

Figure 4 [A, B, and C] illustrates the FTIR spectra for the seed extracts of MP, MP–Se NPs, and CSMNPs, respectively. The absorption peak at 3303 cm^−^¹ corresponds to the stretching vibrations of the hydroxyl group (- OH). A distinct peak at 1635 cm^−^¹ signifies the carbonyl (C = O) stretching. Additionally, the bands at (1540 cm^−^¹, 1473 cm^−^¹, 1458 cm^−^¹, and 1338 cm^−^¹) are due to the stretching vibrations of aromatic C = C and the aromatic C-O group at (1298 cm^−^¹, 1207 cm^−^¹, 1185 cm^−^¹, and 1095 cm^−^¹). The FTIR spectra of CSMNPs displayed distinctive bands due to different functional groups. The absorption band at 3334 cm^−^¹ is the stretching vibration of the hydroxyl group (- OH). At 1634 cm^−^¹, the carbonyl C = O stretch peak was visible in the FTIR spectrum. The aromatic C = C stretch and the aromatic C-O stretch at (1238 cm^−^¹, 1194 cm^−^¹, and 1156 cm^−^¹) are responsible for the bands at (1477 cm^−^¹, 1379 cm^−^¹) in CSMNPs.

**Fig. 5:**
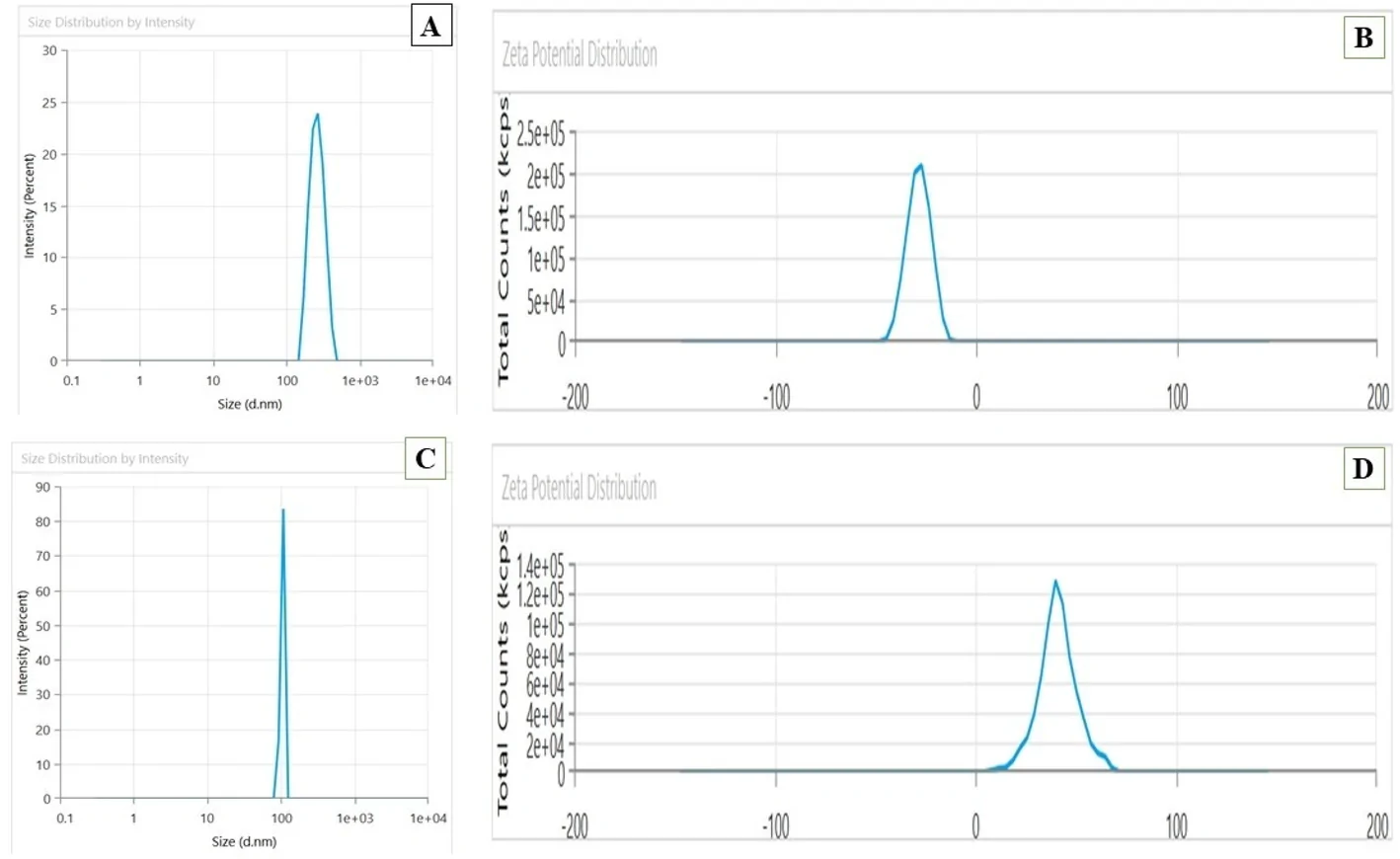
**A** Hydrodynamic diameter of MSNPs; **B** Zeta potential of MSNPs; **C** Hydrodynamic diameter of CSMNPs; **D** Zeta potential of CSMNPs

The hydrodynamic diameter and uniformity of the NP distribution were assessed using the zeta size and polydispersity index. For MSNPs, a low polydispersity index of 0.207 and a size of 278.50 nm confirmed a uniform size distribution (Fig. 5A). The zeta potential of MSNPs was − 28.68 mV, indicating good stability (Fig. 5B). The synthesised CSMNPS showed heterogeneity in distribution with a polydispersity index of 0.977 and a size of 684.6 nm (Fig. 5C). This may be due to interparticle aggregation in aqueous dispersion. The conjugation of the polymer (CS) to MSNPs is responsible for the increased polydispersity. A zeta potential of + 40.91 mV of CSMNPs indicates a highly positively charged surface and a substantial electrostatic repulsion, which may be contributing to colloidal stability (Fig. 5D).

**Fig. 6:**
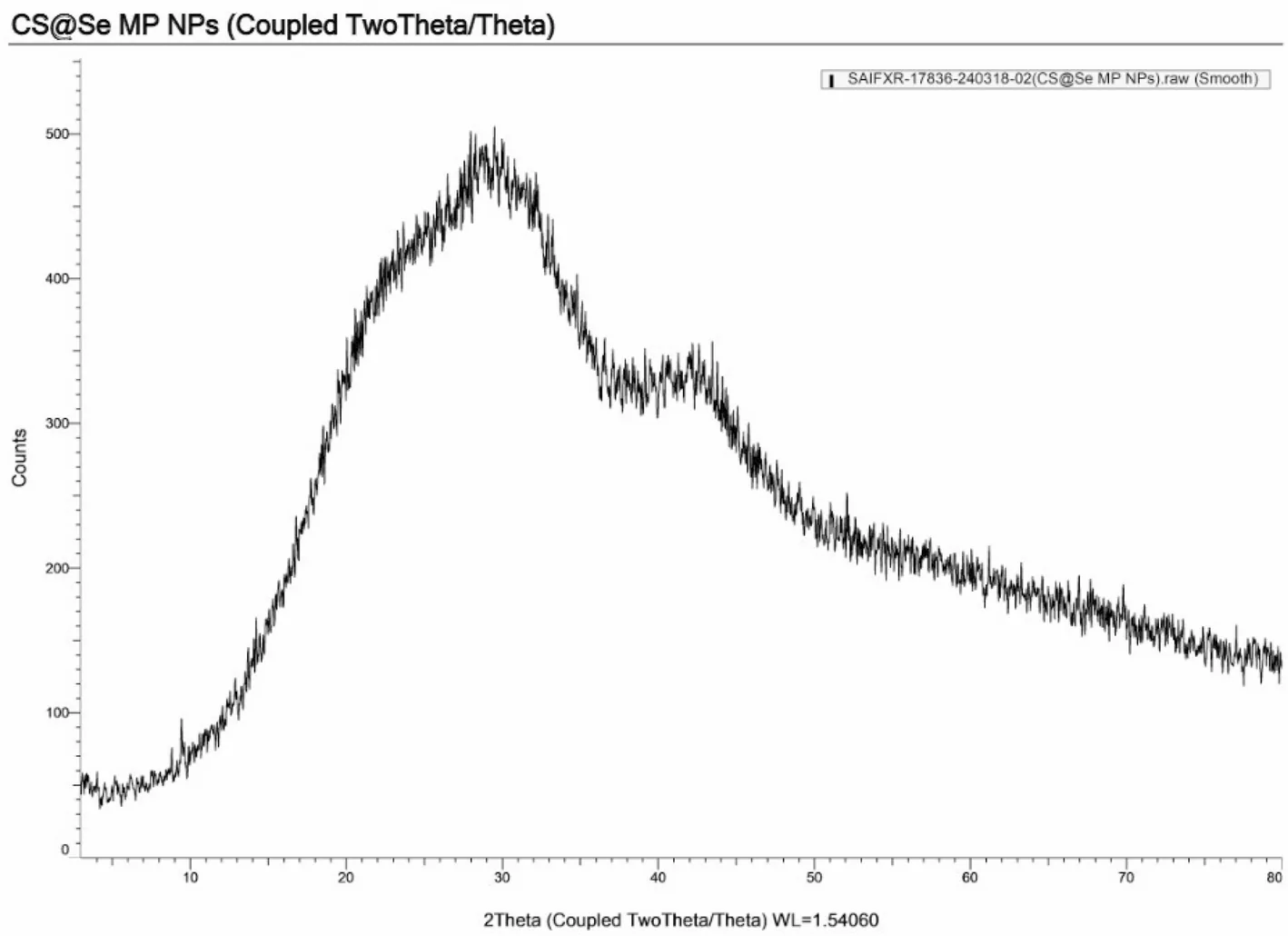
X-ray diffractogram of CSMNPs exhibiting the amorphous property

X-ray diffraction was performed to confirm the physical state of the final nanocomposite CSMNPs. The diffractogram of the CSMNPs does not possess a distinct peak; hence, it is identified as an amorphous material (Fig. 6).

**Fig. 7:**
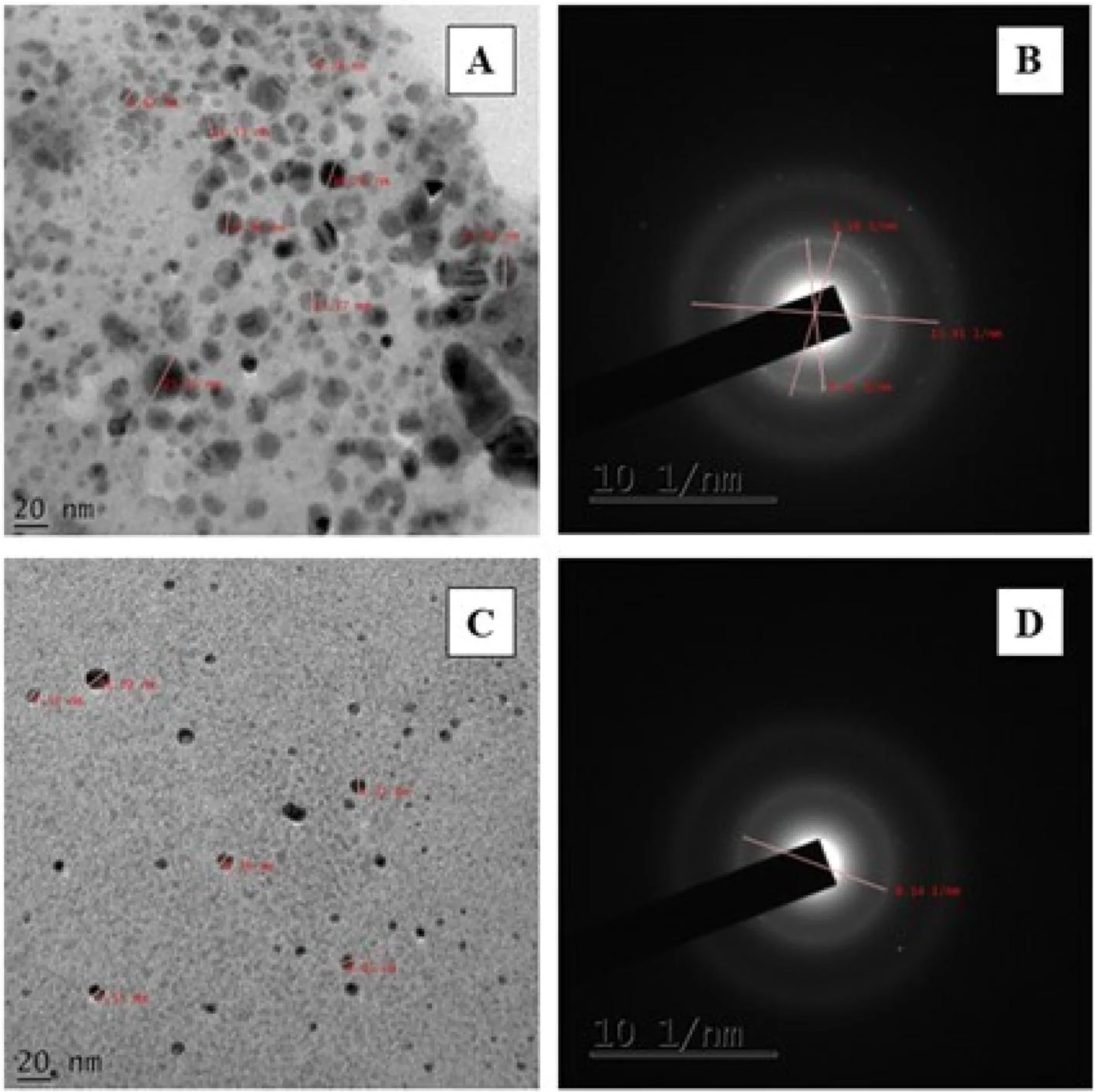
MSNPs and CSMNPs images by HRTEM [**A** and **C**] and the SAED pattern of MSNPs and CSMNPs [**B** and **D**]

MSNPs and CSMNPs were evaluated for size, structure, and morphology using HRTEM with SAED investigations. The spherical NPs in the 9.62–25.76 nm size range were seen in the MSNPs’ HRTEM images (Fig. 7A). Similarly, the synthesised CSMNPs had a spherical shape and ranged in size from 6.71 to 13.22 nm. (Fig. 7C). The polycrystalline characteristics of the MSNPs were indicated by the numerous rings in the SAED pictures. (Fig. 7B). In contrast, the amorphous property of CSMNPs is reflected in Fig. 7D.

### EE and LC of L-DOPA in MSNPs and CSMNPs

The EE (%) and LC (%) of L-DOPA in MSNPs were 75.46 ± 2.48 and 32.15 ± 1.89, respectively. Similarly, the EE (%) and LC (%) in CSMNPs were 87.62 ± 2.56 and 35.85 ± 1.97, respectively. This estimation showcased the efficient entrapment of L-DOPA extracted from MP seeds in the nanoformulations.

### In vitro antioxidant study

**Fig. 8:**
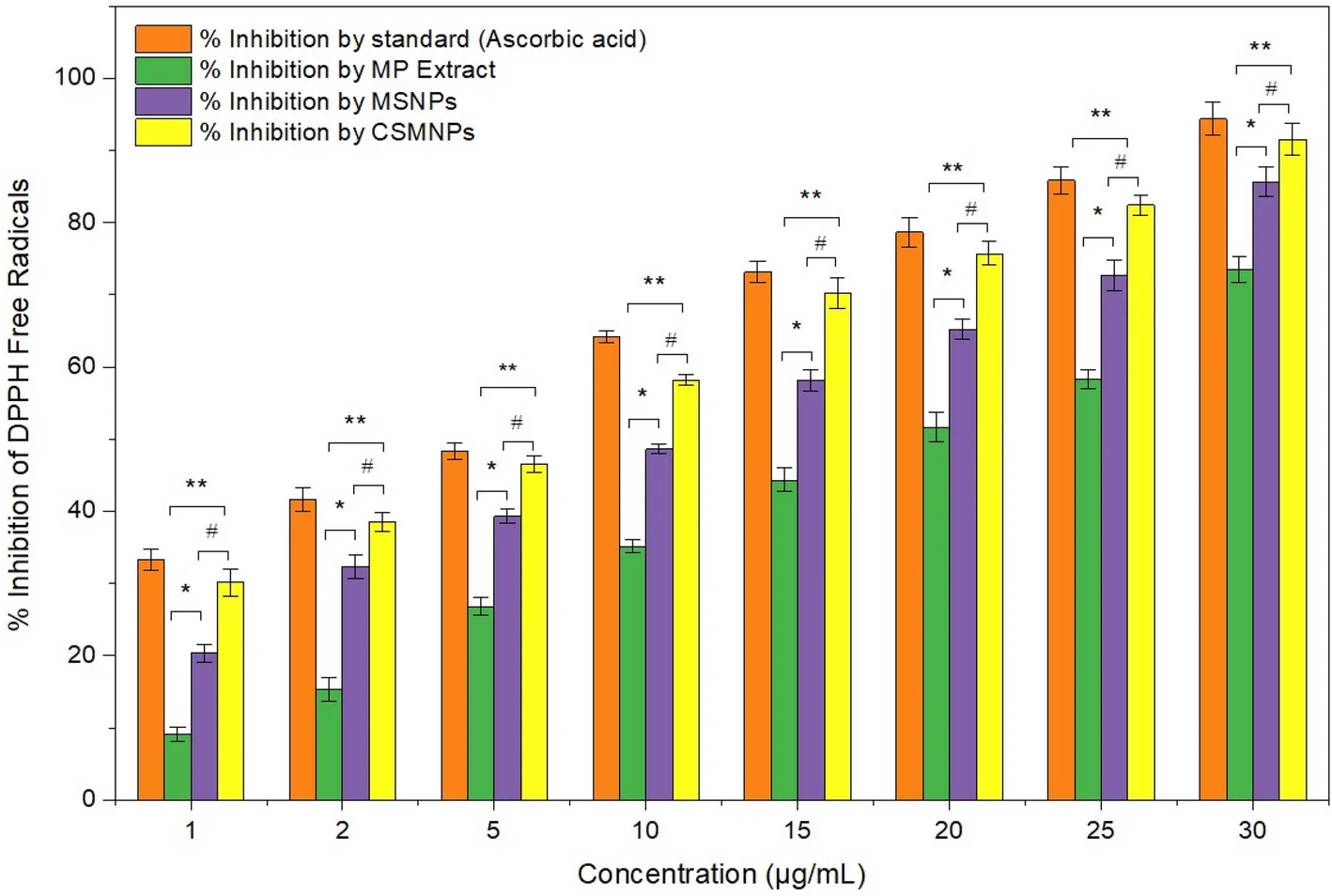
% of inhibition of ascorbic acid, MP extract, MSNPs, and CSMNPs to the DPPH free radical [Each data is expressed as mean ± standard deviation of *n* = 3 for each concentration; Two-way ANOVA followed by Tukey test; *p < 0.05, taken as significant compared with ascorbic acid] *p, where *p < 0.05 for MP Vs MSNPs, ^#^*p* < 0.05 for MSNPs Vs CSMNPs & **p < 0.05 for ascorbic acid Vs CSMNPs]

**Fig. 9:**
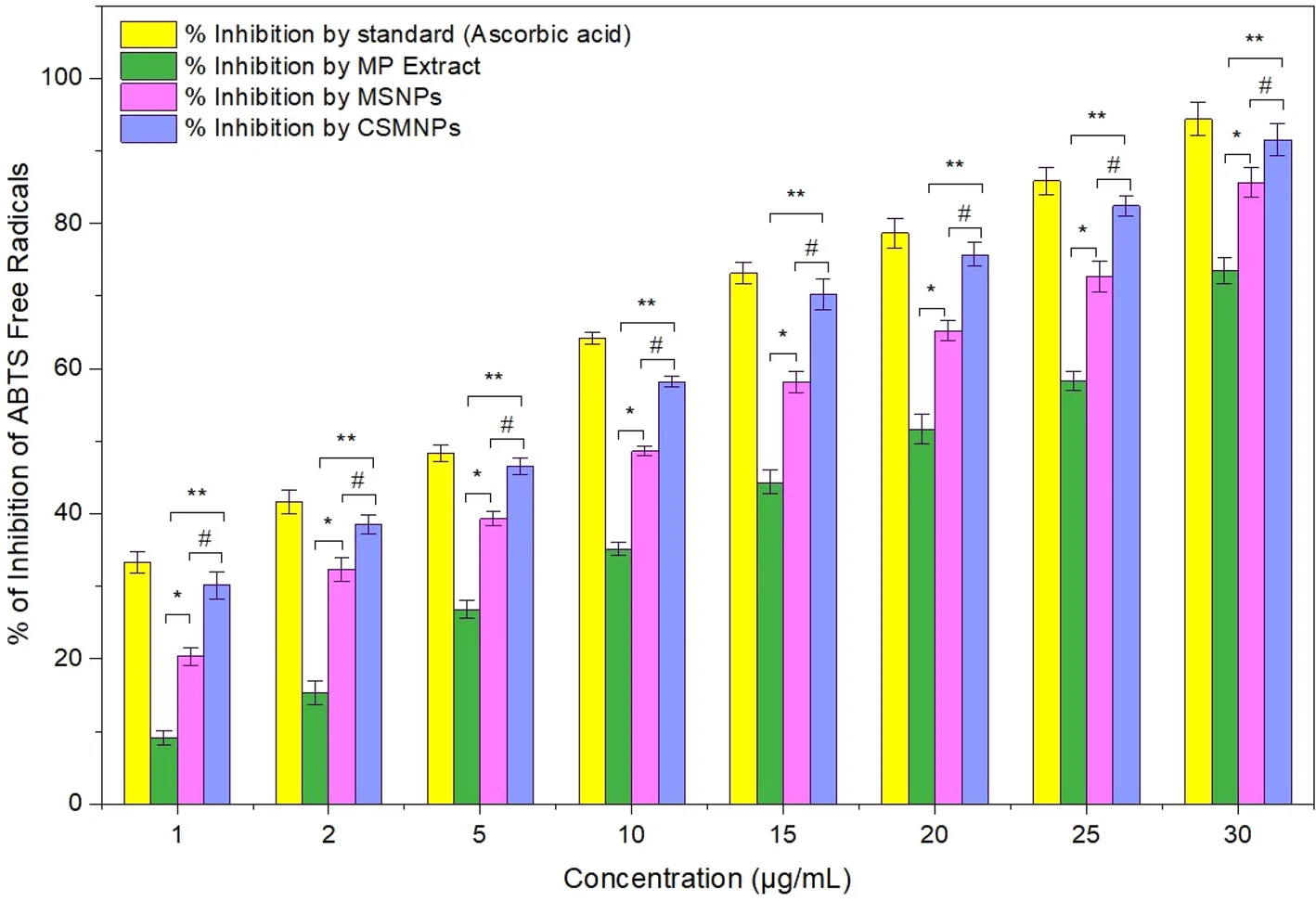
% of inhibition of ascorbic acid, MP extract, MSNPs, and CSMNPs to the ABTS free radical [Each data is expressed as mean ± standard deviation of *n* = 3 for each concentration; Two-way ANOVA followed by Tukey test *p < 0.05, taken as significant compared with ascorbic acid] *p, where *p < 0.05 for MP Vs MSNPs, ^#^*p* < 0.05 for MSNPs Vs CSMNPs & **p < 0.05 for ascorbic acid Vs CSMNPs]

An antioxidant study using the free radical scavenging models DPPH and ABTS was conducted to evaluate the antioxidant potential of MP seed extracts, MSNPs, and CSMNPs by comparing a reference standard (ascorbic acid). The inhibition percentage was assessed and expressed through the IC_50_ value. In this DPPH scavenging assay, 50% inhibitory concentration (IC_50_) values of the standard (ascorbic acid), MP extract, MSNPs, and CSMNPs were 6.25 ± 1.25 µg/mL, 18.94 ± 2.05 µg/mL, 12.61 ± 1.87 µg/mL, and 8.01 ± 1.28 µg/mL, respectively. In the ABTS scavenging assay, the IC_50_ values of ascorbic acid, MP extract, MSNPs, and CSMNPs were 2.35 ± 1.32 µg/mL, 12.85 ± 1.51 µg/mL, 9.29 ± 0.98 µg/mL, and 5.13 ± 1.34 µg/mL, respectively. MP extract, MSNPs, and CSMNPs showed a dose-dependent inhibition of DPPH (Fig. 8) and ABTS (Fig. 9) free radicals. Each data point was processed by one-way ANOVA followed by Tukey’s *t-test*, and the level of significance is expressed at the 5% level.

### Evaluation of neuropharmacological parameters

#### Grip strength test

**Fig. 10:**
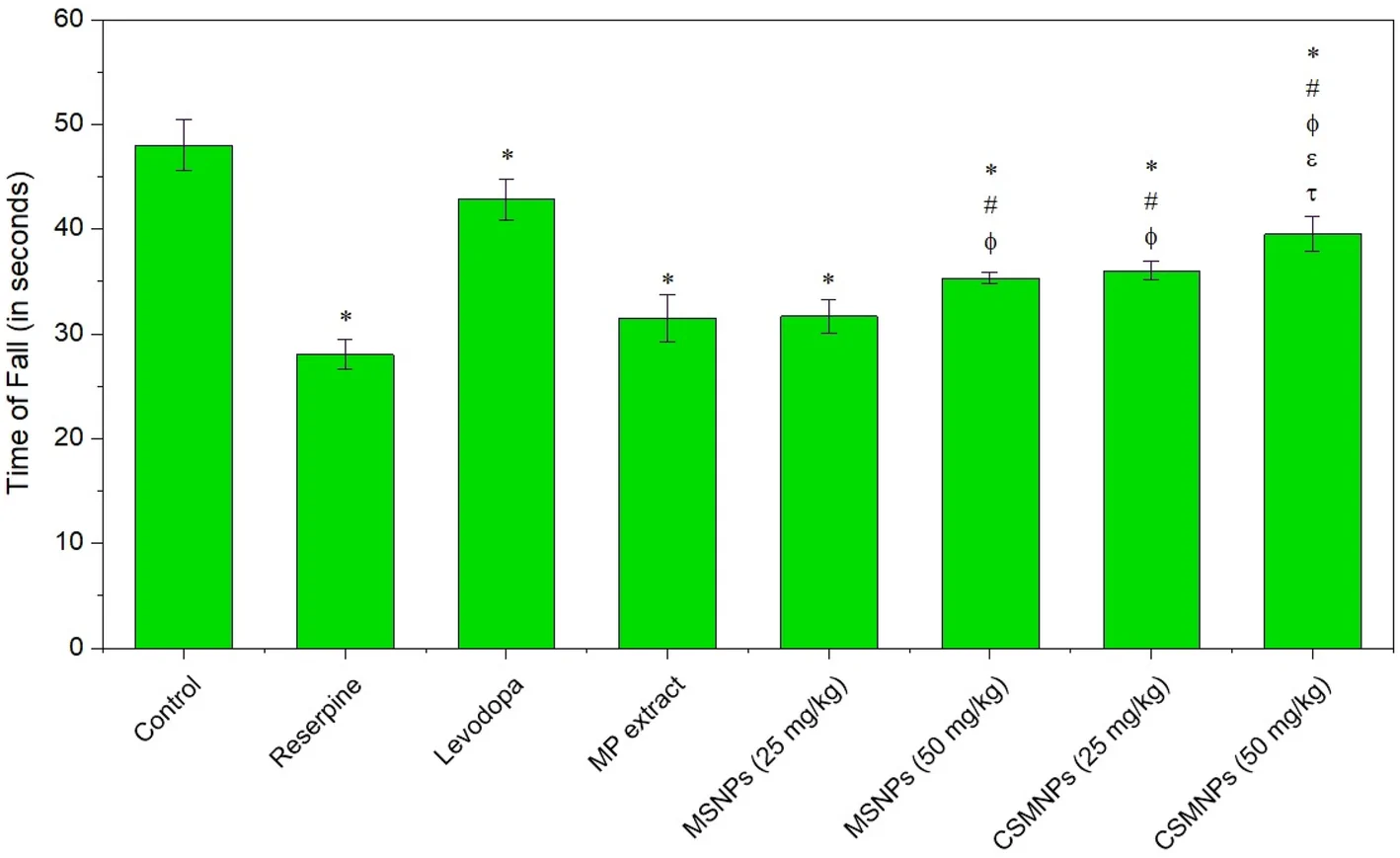
The grip strength of rats in a PD rat model induced by reserpine (0.2 mg•kg^− 1^, i.p.) following administration of MP extract (50 mg•kg^− 1^, p.o.) and MSNPs (25 and 50 mg•kg^− 1^, p.o.) [The values are represented as mean ± Standard deviation; *n* = 6 per group. Tukey’s post hoc t-test was followed by a one-way ANOVA; F(7, 40) ≥ 2.25. *p < 0.05, Reserpine vs. all groups; ^*#*^*p* < 0.05, MP extract vs. MSNPs (50 mg•kg^− 1^) and CSMNPs (25 and 50 mg•kg^− 1^), ^ϕ^*p* < 0.05, MSNPs (25 mg•kg^− 1^), vs. MP NPs (50 mg•kg^− 1^) and CSMNPs (25 and 50 mg•kg^− 1^); ^ε^*p* < 0.05, MSNPs (50 mg•kg^− 1^) vs. CSMNPs (50 mg•kg^− 1^); ^τ^*p* < 0.05, CSMNPs (25 mg•kg^− 1^) vs. CSMNPs (50 mg•kg^− 1^)]

The Rotarod test was conducted using a rat model to assess the grip strength. The induction of reserpine significantly decreased the grip strength. The reserpine-treated rats’ grip strength was substantially lower (*p* < 0.05) than that of the animals in the control group. However, the impact of reserpine (0.2 mg•kg^− 1^) was significantly reversed (*p* < 0.05) by the administration of L-DOPA (100 mg•kg^− 1^), a pure aqueous extract of MP seeds (50 mg•kg^− 1^), MSNPs (25 and 50 mg•kg^− 1^), and CSMNPs (25 and 50 mg•kg^− 1^). No significant difference was found between MP extract and MSNPs (25 mg•kg^− 1^). However, MSNPs (50 mg•kg^− 1^) showed significantly higher grip strength (*p* < 0.05) than both MP extract and MSNPs (25 mg•kg^− 1^). MSNPs (50 mg•kg^− 1^) and CSMNPs (25 mg•kg^− 1^) showed a similar effect to reserpine-induced PD rats. CSMNPs 50 mg•kg^− 1^ showed significantly stronger grip strength (*p* < 0.05) than MP extract, MSNPs (25 and 50 mg•kg^− 1^, and CSMNPs (25 mg•kg^− 1^) (Fig. 10). The statistical significance was further confirmed by Cohen’s d value, which establishes the significance in relation to the group size and its impact on the treatment (Table 1). Each data point is processed by one-way ANOVA followed by Tukey’s *t-test*, and the level of significance is expressed at 5% level.

**Table 1:** Statistical significance in relation to sample size and its effect on the treatment in the grip strength test

Comparison of groups (*n* = 6 for each group)	*p* value	Cohen’s d value	Conclusion
MP Vs MSNPs (25 mg•kg^− 1^)	0.064		Nonsignificant
MP Vs MSNPs (50 mg•kg^− 1^)	0.032	0.86	Significant with a large effect size, indicating a meaningful impact of treatment
MSNPs (25 mg•kg^− 1^) vs. MSNPs (50 mg•kg^− 1^)	0.021	1.08	
MP Vs CSMNPs (25 mg•kg^− 1^)	0.045	0.93	
MP Vs CSMNPs (50 mg•kg^− 1^)	0.024	0.91	
MSNPs (25 mg•kg^− 1^) vs. CSMNPs (25 mg•kg^− 1^)	0.019	1.38	
MSNPs (50 mg•kg^− 1^) vs. CSMNPs (25 mg•kg^− 1^)	0.037	1.21	
MSNPs (25 mg•kg^− 1^) vs. CSMNPs (50 mg•kg^− 1^)	0.004	0.87	
MSNPs (50 mg•kg^− 1^) vs. CSMNPs (50 mg•kg^− 1^)	0.012	1.54	
CSMNPs (25 mg•kg^− 1^) Vs CSMNPs (50 mg•kg^− 1^)	0.036	0.98	

#### Catalepsy test

**Fig. 11:**
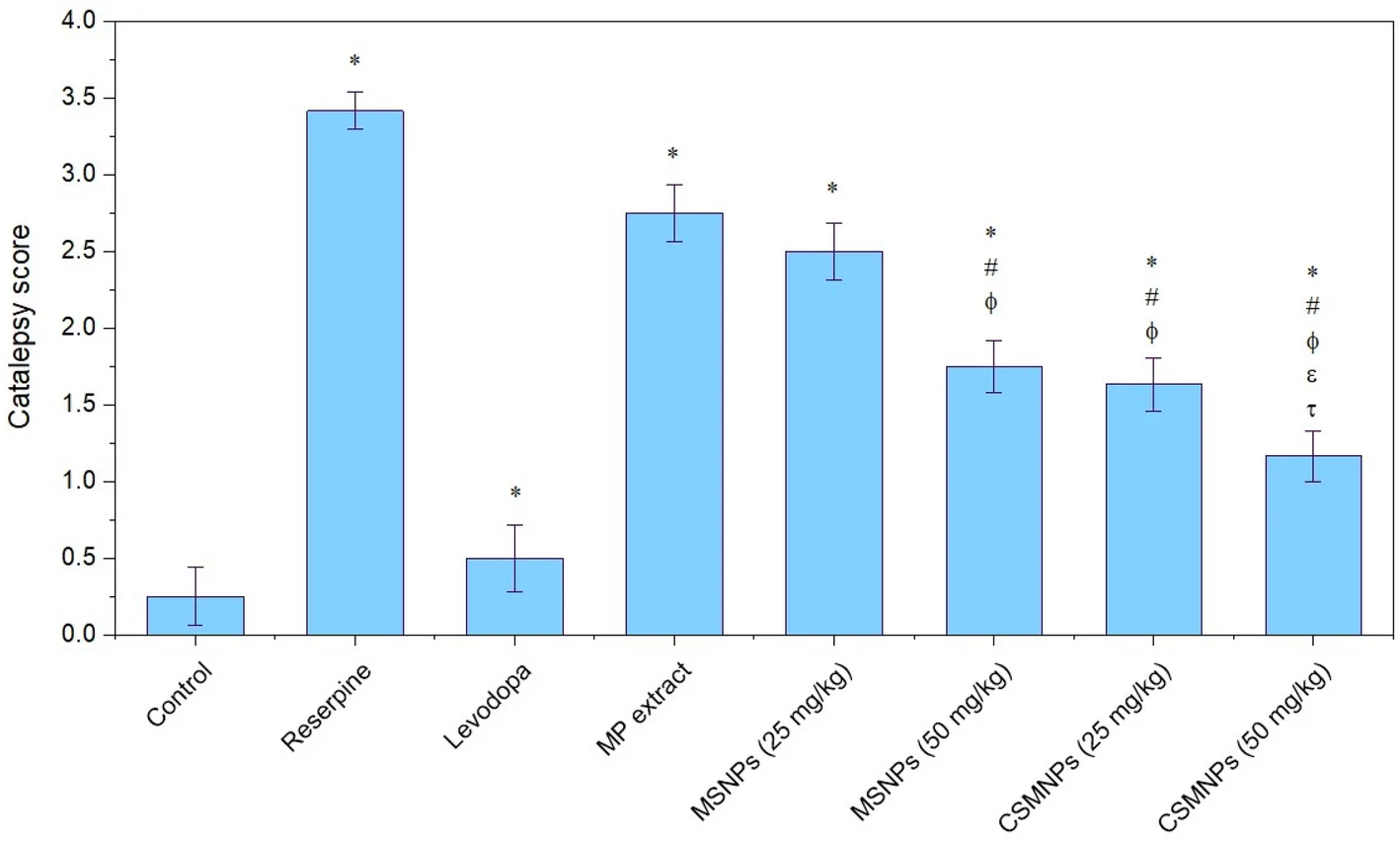
Cataleptic score of rats after administration of MP extract (50 mg•kg^− 1^, p.o.) and MSNPs (25 and 50 mg•kg^− 1^, p.o.) on reserpine (0.2 mg•kg^− 1^, i.p.) induced PD rat model [The values are represented as mean ± Standard deviation; *n* = 6 per group. Tukey’s post hoc t-test was followed by a one-way ANOVA; F(7, 40) ≥ 2.25.*p < 0.05, Reserpine vs. all groups; ^*#*^*p* < 0.05, MP extract vs. MSNPs (50 mg•kg^− 1^) and CSMNPs (25 and 50 mg•kg^− 1^); ^ϕ^*p* < 0.05, MSNPs (25 mg•kg^− 1^) vs. MSNPs (50 mg•kg^− 1^) and CSMNPs (25 and 50 mg•kg^− 1^); ^ε^*p* < 0.05, MSNPs (50 mg•kg^− 1^) vs. CSMNPs (50 mg•kg^− 1^); ^τ^*p* < 0.05, CSMNPs (25 mg•kg^− 1^) vs. CSMNPs (50 mg•kg^− 1^)]

The catatonic response was tested on a wooden block. The rats treated with reserpine experienced significantly higher cataleptic scores (*p* < 0.05) than the control group. However, reserpine’s effect (0.50 mg•kg^− 1^) was reversed considerably (*p* < 0.05) by administration of L-DOPA (100 mg•kg^− 1^), pure aqueous extract of MP seeds (50 mg•kg^− 1^), MSNPs (25 and 50 mg•kg^− 1^), and CSMNPs (25 and 50 mg•kg^− 1^). No significant difference was found between MP extract and MSNPs (25 mg•kg^− 1^). MSNPs (50 mg•kg^− 1^) showed a significantly lower catatonic response (*p* < 0.05) than both MP extract and MSNPs (25 mg•kg^− 1^). MSNPs (50 mg•kg^− 1^) and CSMNPs (25 mg•kg^− 1^) showed a similar effect to reserpine-induced PD rats. CSMNPs (50 mg•kg^− 1^) showed a significantly lower catatonic response (*p* < 0.05) than MP extract, MSNPs (25 and 50 mg•kg^− 1^, and CSMNPs (25 mg•kg^− 1^) (Fig. 11). The statistical significance was further confirmed by Cohen’s d value, which establishes the significance in relation to the group size and its impact on the treatment (Table 2). Each data point was processed by one-way ANOVA followed by Tukey’s *t-test*, and the level of significance is expressed at 5% level.

**Table 2:** Statistical significance in relation to sample size and its effect on the treatment in the catalepsy test

Comparison of groups (*n* = 6 for each group)	*p* value	Cohen’s d value	Conclusion
MP Vs MSNPs (25 mg•kg^− 1^)	0.087		Nonsignificant
MP Vs MSNPs (50 mg•kg^− 1^)	0.0452	1.43	Significant with a large effect size, indicating a meaningful impact of treatment
MSNPs (25 mgkg^− 1^) Vs MSNPs (50 mg•kg^− 1^)	0.0119	0.89	
MP Vs CSMNPs (25 mg•kg^− 1^)	0.0214	1.23	
MP Vs CSMNPs (50 mg•kg^− 1^)	0.0208	1.28	
MSNPs (25 mg•kg^− 1^) vs. CSMNPs (25 mg•kg^− 1^)	0.0347	0.82	
MSNPs (50 mg•kg^− 1^) vs. CSMNPs (25 mg•kg^− 1^)	0.0304	1.84	
MSNPs (25 mg•kg^− 1^) vs. CSMNPs (50 mg•kg^− 1^)	0.016	1.08	
MSNPs (50 mg•kg^− 1^) vs. CSMNPs (50 mg•kg^− 1^)	0.028	1.19	
CSMNPs (25 mg•kg^− 1^) Vs CSMNPs (50 mg•kg^− 1^)	0.035	1.61	

### In vivo antioxidant studies

#### SOD levels estimation

**Fig. 12:**
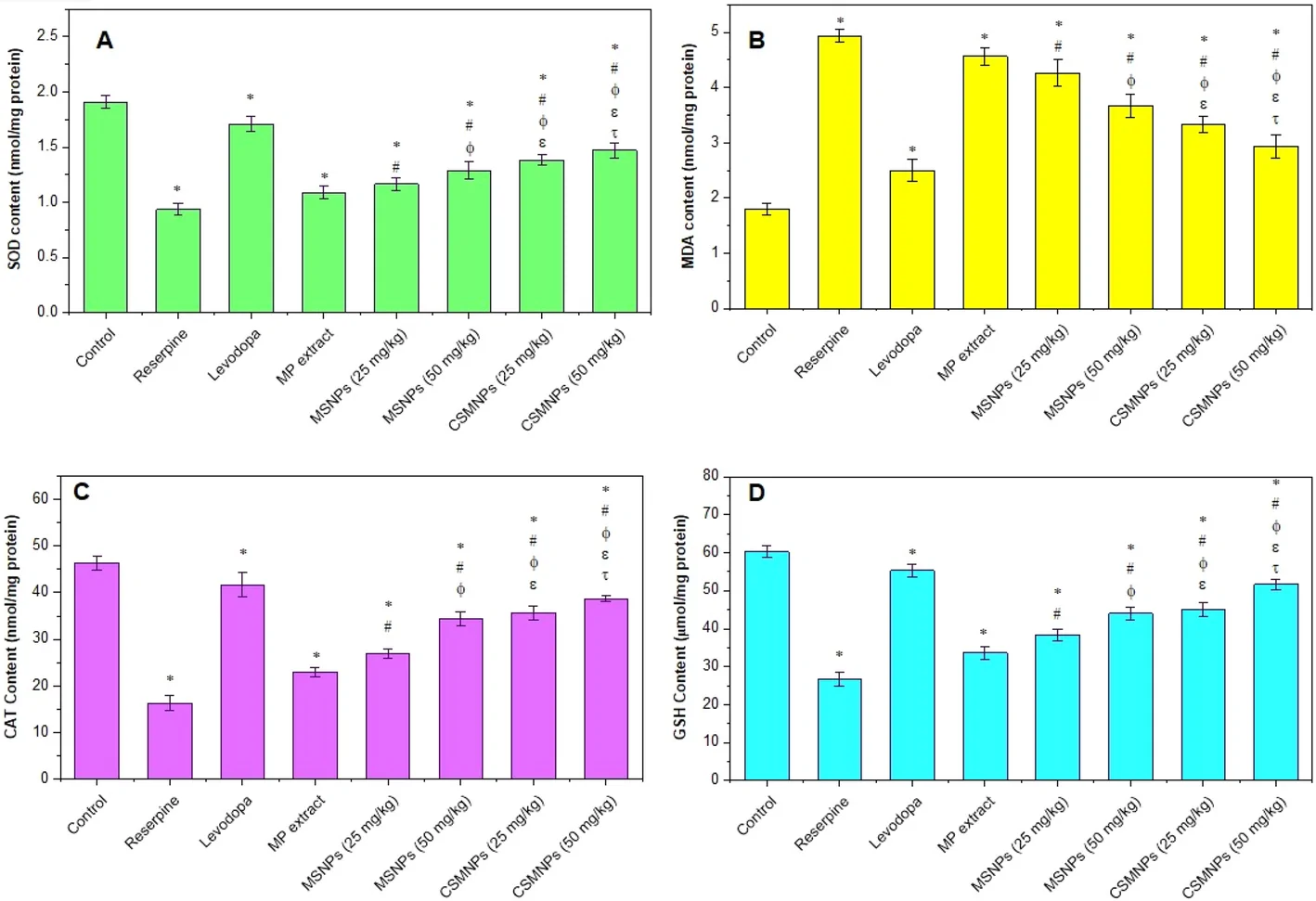
Content of SOD [**A**], MDA [**B**], CAT [**C**], and GSH [**D**] of rats after administration of MP extract (50 mg•kg^− 1^, p.o.) and MSNPs (25 and 50 mg•kg^− 1^, p.o.) on reserpine (0.2 mg•kg^− 1^, i.p.) induced PD rat model. [The values are represented as mean ± Standard deviation; *n* = 3 per group. Tukey’s post hoc t-test was followed by a one-way ANOVA; F(7, 16) ≥ 2.25. *p < 0.05, Reserpine vs. all groups; ^*#*^*p* < 0.05, MP extract vs. MSNPs (50 mg•kg^− 1^) and CSMNPs (25 and 50 mg•kg^− 1^); ^ϕ^*p* < 0.05, MSNPs (25 mg•kg^− 1^) vs. MSNPs (50 mg•kg^− 1^) and CSMNPs (25 and 50 mg•kg^− 1^); ^ε^*p* < 0.05, MSNPs (50 mg•kg^− 1^) vs. CSMNPs (50 mg•kg^− 1^); ^τ^*p* < 0.05, CSMNPs (25 mg•kg^− 1^) vs. CSMNPs (50 mg•kg^− 1^)]

An important enzyme is SOD, as it reduces the production of reactive oxygen species, thereby helping to limit cellular damage. By transforming the harmful superoxide anion into less harmful H_2_O_2_ and oxygen ions, it serves as a defence mechanism against oxidation-induced stress. SOD and catalase are both defensive enzymes that detoxify extremely reactive free radicals, which have a comparable function. In contrast to the control group, reserpine induction significantly decreases SOD content (*p* < 0.05). However, the effect of reserpine was dramatically reversed. (*p* < 0.05) by administration of L-DOPA (100 mg•kg^− 1^), MP pure aqueous seed extract (50 mg•kg^− 1^), and MSNPs (25 mg•kg^− 1^ and 50 mg•kg^− 1^). No significant difference was found between MP extract and MSNPs (25 mg•kg^− 1^). MSNPs (50 mg•kg^− 1^) showed significantly higher SOD content (*p* < 0.05) than both MP extract and MSNPs (25 mg•kg^− 1^). MSNPs (50 mg•kg^− 1^) and CSMNPs (25 mg•kg^− 1^) showed a similar effect to reserpine-induced PD rats, and CSMNPs (50 mg•kg^− 1^) showed significantly more SOD content (*p* < 0.05) than MP extract, MSNPs (25 and 50 mg•kg^− 1^, and CSMNPs (25 mg•kg^− 1^) (Fig. 12A). Each data point is processed by one-way ANOVA followed by Tukey’s *t-test*, and the level of significance is expressed at the 5% level.

#### Determination of MDA (extent of lipid peroxidation)

In PD, the escalated production of reactive oxygen species (ROS) leads to oxidative stress, a primary cause of cellular damage. The primary target of ROS is the acceleration of oxidation of lipids in the brain. The increase in MDA concentration weakens SOD and other antioxidant enzymes’ defence mechanisms. MDA content increased (*p* < 0.05) in the reserpine-induced PD rat. However, reserpine’s effect was significantly reversed (*p* < 0.05) by administration of L-DOPA (100 mg•kg^− 1^), pure aqueous seed extract of MP (50 mg•kg^− 1^), and MSNPs (25 mg•kg^−1^and 50 mg•kg^− 1^). No significant difference was found between MP extract and MSNPs (25 mg•kg^− 1^). MSNPs (50 mg•kg^− 1^) showed a significantly lower MDA level (*p* < 0.05) than both MP extract and MSNPs (25 mg•kg^− 1^). MSNPs (50 mg•kg^− 1^) and CSMNPs (25 mg•kg^− 1^) showed a similar effect to reserpine-induced PD rats, and CSMNPs (50 mg•kg^− 1^) showed markedly lower MDA levels (*p* < 0.05) than MP extract, MSNPs (25 and 50 mg•kg^− 1^, and CSMNPs (25 mg•kg^− 1^) (Fig. 12B). Each data point is processed by one-way ANOVA followed by Tukey’s *t-test*, and the level of significance is expressed at 5% level.

#### Determination of CAT content

For the breakdown of hydrogen peroxide (H_2_O_2_) ions into water and oxygen, catalase is the most crucial enzyme reported to detoxify the generated SOD. When reserpine is administered, the catalase level is drastically decreased, leading to motor impairment. Compared to the control group, induction of reserpine significantly deteriorates CAT content (*p* < 0.05) in PD-induced rats. However, the effect of reserpine was significantly reversed (*p* < 0.05) by administration of L-DOPA (100 mg•kg^− 1^), MP aqueous seed extract (50 mg•kg^− 1^), and MSNPs (25 mg•kg^−1^and 50 mg•kg^− 1^). No significant difference was found between MP extract and MSNPs (25 mg•kg^− 1^). MSNPs (50 mg•kg^− 1^) showed significantly higher CAT content (*p* < 0.05) than both MP extract and MSNPs (25 mg•kg^− 1^). MSNPs (50 mg•kg^− 1^) and CSMNPs (25 mg•kg^− 1^) showed a similar effect to reserpine-induced PD rat, and CSMNPs (50 mg•kg^− 1^) showed significantly increased CAT content (*p* < 0.05) than MP extract, MSNPs (25 and 50 mg•kg^− 1^, and CSMNPs (25 mg•kg^− 1^) (Fig. 12C). Each data point is processed by one-way ANOVA followed by Tukey’s *t-test*, and the level of significance is expressed at 5% level.

#### Determination of GSH

Reduced glutathione acts as the substrate for the glutathione peroxidase enzyme. It generates the defence mechanism against free radicals directly. As compared to the control, reserpine induction considerably lowers GSH levels (*p* < 0.05). However, the effect of reserpine was reversed (*p* < 0.05) by the administration of L-DOPA (100 mg•kg^− 1^), pure aqueous seed extract of MP (50 mg•kg^− 1^), and MSNPs (25 mg•kg^−1^and 50 mg•kg^− 1^). No significant difference was found between MP extract and MSNPs (25 mg•kg^− 1^). MSNPs (50 mg•kg^− 1^) showed significantly higher GSH levels (*p* < 0.05) than both MP extract and MSNPs (25 mg•kg^− 1^). MSNPs (50 mg•kg^− 1^) and CSMNPs (25 mg•kg^− 1^) showed a similar effect to reserpine-induced PD rats. But the GSH content of CSMNPs (50 mg•kg^− 1^) was noticeably higher (*p* < 0.05) than MP extract, MSNPs (25 and 50 mg•kg^− 1^, and CSMNPs (25 mg•kg^− 1^) (Fig. 12D). Each data point was processed by one-way ANOVA followed by Tukey’s *t-test*, and the level of significance was expressed at the 5% level.

### Estimation of nitrite level

**Fig. 13:**
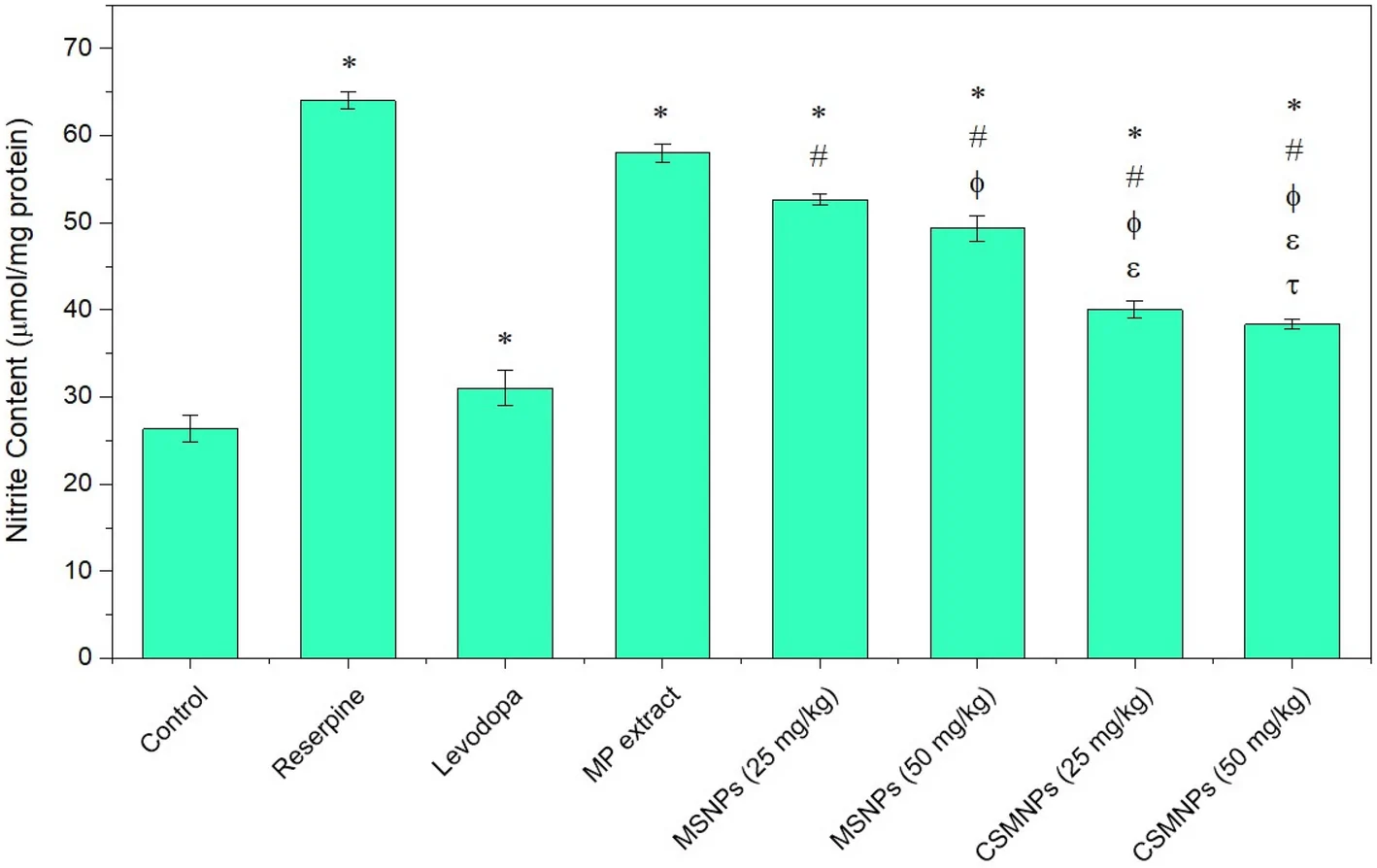
Content of nitrite in rats after administration of MP extract (50 mg•kg^-1^, p.o.) and MSNPs (25 and 50 mg•kg^-1^, p.o.) on reserpine (0.2 mg•kg^-1^, i.p.) induced PD rat model. [The values are represented as mean ± Standard deviation; *n* = 3 per group. .Tukey’s post hoc t-test was followed by a one-way ANOVA; F(7, 16) ≥ 2.25. *p < 0.05, Reserpine vs. all groups; ^*#*^*p* < 0.05, MP extract vs. MSNPs (50 mg•kg^-1^) and CSMNPs (25 and 50 mg•kg^-1^); ^ϕ^*p* < 0.05, MSNPs (25 mg•kg^-1^) vs. MSNPs (50 mg•kg^-1^) and CSMNPs (25 and 50 mg•kg^-1^); ^ε^*p* < 0.05, MSNPs (50 mg•kg^-1^) vs. CSMNPs (50 mg•kg^-1^); ^τ^*p* < 0.05, CSMNPs (25 mg•kg^-1^) vs. CSMNPs (50 mg•kg^-1^)]

In the reserpine-induced PD rat, nitrite concentration increased significantly (*p* < 0.05); however, L-DOPA (100 mg•kg^− 1^), pure aqueous seed extract of MP (50 mg•kg^− 1^), and MSNPs (25 mg•kg^− 1^ and 50 mg•kg^− 1^) effectively reversed the impact of reserpine (*p* < 0.05). There was no discernible difference between MP extract and MSNPs (25 mg•kg^− 1^). The nitrite concentration of MSNPs (50 mg•kg^− 1^) was substantially lower (*p* < 0.05) than that of MP extract and MSNPs (25 mg•kg^− 1^). Compared to MP extract, MSNPs (25 and 50 mg•kg^− 1^), and CSMNPs (25 mg•kg^− 1^), CSMNPs (50 mg•kg^− 1^) showed a considerably decreased nitrite content (*p* < 0.05), and MSNPs (50 mg•kg^− 1^) and CSMNPs (25 mg•kg^− 1^) had an effect comparable to reserpine-induced PD rats (Fig. 13). Each data point is processed by one-way ANOVA followed by Tukey’s *t-test*, and the level of significance is expressed at 5% level.

### Histopathological study of hippocampus

**Fig. 14:**
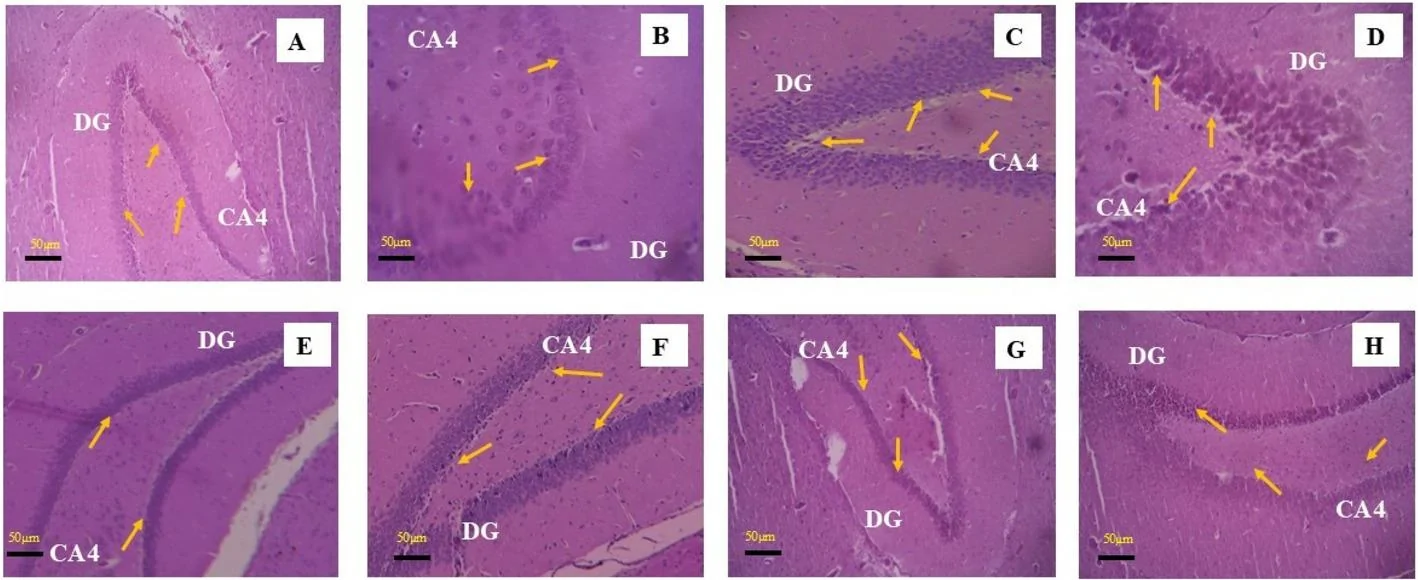
Histopathological representation showing the *Cornu Ammonis* (CA4) and the dentate gyrus (DG) region of the hippocampus of the brain of Group I-VI (scale = 100 μm, 10x magnification); **A** represents the control group - regular neuroglial cell line structure; **B** Reserpine treatment group: the hippocampus area exhibiting severe necrosis, pyknosis, and neuroglial cell degeneration; **C** As shown, the L-DOPA group appears normal with fewer cases of pyknosis and necrosis. The cells with reduced neurodegeneration, less necrosis, and pyknosis are represented by [**D**, **E**, **F**, **G**, **H**] for MP extract, MSNPs (25 mg•kg^− 1^), MSNPs (50 mg•kg^− 1^), and CSMNPs (25 and 50 mg•kg^− 1^) groups, respectively

The histopathological examination showed less neurodegeneration in the dentate gyrus (DG) and cornu ammonis (CA4) of the hippocampus in reserpine-induced PD rats. Neuronal cells in the control group were well-organised. Neuroglial cells exhibited pyknotic characteristics, which is a normal finding. On the other hand, severe pyknotic and necrotic signs were seen in the hippocampal neurons of reserpine-administered rats, suggesting widespread neurodegeneration and partial loss of the neuronal population, which was not the situation in the control group. On the other hand, the treatment with MP extract, MSNPs (25 mg•kg^− 1^), MSNPs (50 mg•kg^− 1^) and CSMNPs (25 and 50 mg•kg^− 1^) appeared to have a protective role when compared to the reserpine-treated group (Fig. 14A, B, C, D, E, F, G, H).

### Histopathology of Substantia nigra of the midbrain

**Fig. 15:**
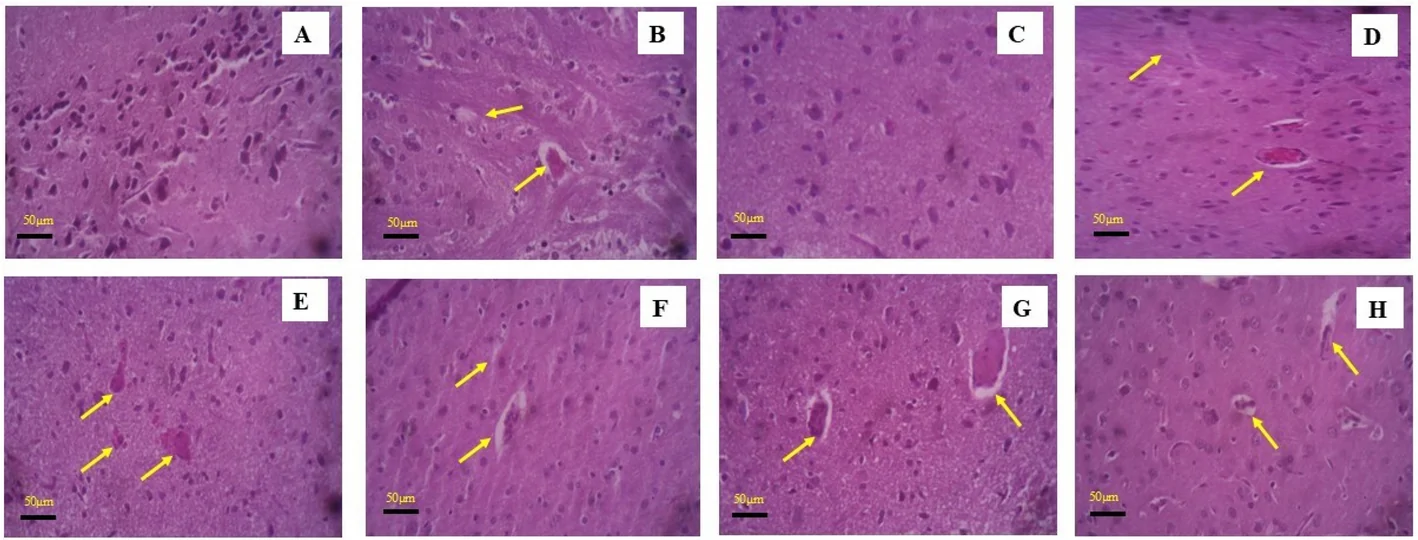
Histopathological representation showing the substantia nigra of the brain of Group I-VI (scale = 100 μm; 10x magnification); **A** stands for control group-normal neuroglial cell line; **B** Reserpine group demonstrating neurons with collapsed, hyperchromatic, deep basophilic nuclei, aberrant Nissl granule distribution, and encircled by either deep eosinophilic cytoplasm or a halo; additionally, dark, dense granules known as incrustations can be observed forming on or near the surface of neurons. **C** The L-DOPA group displayed some tissue structural improvements. Nissl granules were scattered and luminous; vesicular, conspicuous nuclei were seen in normal neurons; blood capillaries were found in a normal assembly with a few congestions (wave arrow); **D**, **E**, **F**, **G**, **H** represent MP extract, MSNPs (25 mg•kg^− 1^), MSNPs (50 mg•kg^− 1^), and CSMNPs (25 and 50 mg•kg^− 1^) groups, respectively, showing better tissue structure, prominent nuclei, and dispersed Nissl granules

Histopathological results showed that the substantia nigra compacta of the PD rat model exhibited substantial loss and death of dopaminergic neurons, as well as necrosis and shrinkage. Normal neuronal assembly (light vesicular nuclei, prominent nucleoli) and peripheral distribution of Nissl granules were seen in group I. The rats treated with reserpine displayed a remarkable loss of neurons, accompanied by the accumulation of intracytoplasmic Lewis bodies (yellow arrows). Protective effects were observed in rats treated with MP extract, MSNPs (25 mg•kg^− 1^), MSNPs (50 mg•kg^− 1^), and CSMNPs (25 and 50 mg•kg^− 1^) as compared to the reserpine-treated group (Fig. 15A, B, C, D, E, F, G, H).

## Discussion

Se NPs are explored for their efficacy in PD due to their antioxidant properties. Selenium is a vital trace element found in selenium-containing proteins, such as glutathione peroxidases, which neutralise harmful free radicals and superoxide radicals and cause a reduction in oxidative stress. Se NPs may enhance the antioxidant defence mechanisms in the brain associated with PD [7]. The improved activity observed with MSNPs in the present study may be associated with the ability of Se to support endogenous antioxidant defence and reduce oxidative imbalance, which is an important component of reserpine-induced neurotoxicity. In addition, the association of Se with the bioactive constituents of MP may provide a complementary antioxidant effect, potentially contributing to the observed antiparkinsonian activity. So, we choose to synthesise Se NPs of aqueous extract of *MP* seeds using a chemical reduction technique and to study their efficacy in reserpine-induced catalepsy and reserpine-induced PD in rats.

CS is one of the most widely utilised and safe natural biopolymers in the pharmaceutical sector. It comes from the universe of nature. It is a polycationic, non-toxic stabilising agent used to prepare polymeric NPs. The negative surface of the cell membrane may also attract particles with a positive surface charge [72]. Aggregation would result from the van der Waals forces between bare metallic NPs without an appropriate stabiliser. On the other hand, due to significant electrostatic repulsion, CS, a steric barrier that envelops the metal with a positive charge, produces a homogeneous NP solution [73, 74]. It has also been confirmed that CS could serve as a good stabiliser for Se-NPs. Due to the potential antioxidant activity of CS [75], the in vitro antioxidant properties of CSMNPs were significantly enhanced [76]. Thus, the incorporation of CS may have contributed to the improved stability and biological performance of the Se-based nano formulation observed in the present study.

To show an effect on the central nervous system, a drug should be permeable through the BBB. A nano formulation is a widely used technique to increase BBB permeability. They can effectively pass the BBB due to their tiny dimensions, enhanced stability, and increased drug-loading capacity. A study on Se NPs reported their enhanced bioavailability and easy permeability through the BBB [77]. Again, nanocomposites designed by tailoring polymers to the surface of metal NPs not only increase stability but also enhance bioavailability, efficacy, and potency. The present study was designed to evaluate the antiparkinsonian activity of Se NPs conjugated with MP and CS. The hydrophobic component of CS derivatives facilitates the release of anionic ions that are bonded to CS by the formation of polyplexes. Additionally, the hydrophobic moieties enhance cellular adhesion on neural cell surfaces [78]. The investigation began with extraction using a green technology, such as microwave-assisted extraction [79, 80] and characterising the aqueous extract of MP seed powder. This was followed by the synthesis and characterisation of metal NPs (MSNPs). We have synthesised and characterised polymeric-metal nanocomposites (CSMNPs) to stabilise Se NPs. Furthermore, the study included an antioxidant assay using both in vitro and in vivo models, as well as an evaluation of antiparkinsonian activity. In our experimental design, we used two different concentrations of MSNPs and CSMNPs (25 mg•kg^− 1^) and (50 mg•kg^− 1^) to prevent the neurotoxic effect of Se in NPs form. Lieu et al. (2010) reported the antiparkinsonian efficacy of the aqueous extract of MP seed powder at two dose levels: 12 mg•kg^− 1^ and 20 mg•kg^− 1^ [81]. In our research, the L-DOPA content was found to be 27.5% in MP seeds through microwave-assisted extraction, compared to earlier reports of 5–7% L-DOPA in MP seeds obtained by traditional extraction methods. Hence, as compared to L-DOPA (100 mg•kg^− 1^), we chose to use half of the dose for the seed extract of MP, i.e., 50 mg•kg^− 1^. The results obtained with lower doses of Se NPs and CS@Se NPs were comparable to those of L-DOPA (100 mg•kg^− 1^) and MP extract (50 mg•kg^− 1^). All the studies are statistically analysed for the significant neuroprotective and antioxidant activity of MSNPs and CSMNPs.

To confirm the synthesis and characterise the MSNPs and CSMNPs, various analytical methods were employed, including ultraviolet-visible spectroscopy, FTIR spectroscopy, hydrodynamic diameter, zeta potential, and HRTEM-SAED. The SPR of MSNPs and CSMNPs was used to confirm the synthesis of Se NPs by observing the absorbance and comparing it with the literature [7]. The FTIR study was conducted to understand the interaction between functional groups in the reduction and stabilisation of metal NPs. The stretching and bending vibrations of the MSNPs and CSMNPs were compared with those of the MP extract [80]. A zeta sizer confirmed the hydrodynamic diameter of the NPs, and the determination of zeta potential showcased the stability of the metal NPs. The polydispersity index (PDI) value represented the dispersion of the NPs and the size distribution [54]. Surface functionalisation of MSNPs with chitosan, resulting in CSMNPs, increased the hydrodynamic diameter due to possible interparticle association and aggregation. The zeta potential determined the colloidal stability of the MSNPs, and the conjugation of CS with MSNPs, forming CSMNPs, indicated a higher surface positive charge due to possible electrostatic repulsion between NPs. The HRTEM investigation showed the spherical shape of MSNPs and CSMNPs. The differences in the hydrodynamic diameter and the size by HRTEM are substantially different due to the different principles involved in the measurement. In the measurement of hydrodynamic diameter, the measurement of the NPs involved the hydrated polymer layer and aggregation of NPs in dispersion. Whereas, in HRTEM, the measurement of size involved the measurement of dried and deposited primary NPs on the TEM grid. Hence, it also implied that the crystallinity properties of the NPs and the CSMNPs were confirmed to be amorphous from the XRD study.

Free radical scavenging models, such as the DPPH and ABTS assays, were employed to investigate the antioxidant properties using an in vitro method [63]. It was found that the absorbance of the DPPH free radical decreased gradually with an increase in concentration, as confirmed by observing the colour change compared to ascorbic acid at λ_max_ = 515 nm. The results revealed that the MSNPs and CSMNPs transfer electrons to the DPPH^•^ free radical (purple) and transform it into stable DPPH-H (yellow). Similarly, in the presence of MSNPs and CSMNPs, the ABTS^•+^ (dark green colour) was reduced to ABTS (colourless). As the concentration of NPs increased, the free radical reduction ability also increased. The inhibitory concentration at 50% (IC_50_) of MP extract, MSNPs, and CSMNPs was significantly higher than that of ascorbic acid.

The neuropharmacological analysis generally evaluates the drug’s effect on the CNS. The primary challenges regarding the pharmacological and neural mechanisms of complex behaviour and the irregularities observed in neurological disorders are related to behavioural neuropharmacological activities [61]. The grip strength test and catalepsy score were evaluated to assess neurobehavioral activity. The grip strength or rotarod test is used to assess the effect of a drug on neuromuscular motor coordination. A wooden block was used to evaluate the grip strength. Reserpine administration significantly reduced grip strength in rats, indicating impaired neuromuscular coordination associated with Parkinsonian symptoms, which is aligned with previous studies [61]. Treatment with L-DOPA, MP extract, MSNPs, and CSMNPs improved grip strength compared with the reserpine-induced PD rat group. The comparatively better motor performance observed with the higher dose of MSNPs and CSMNPs suggests that the nanoparticle formulations were able to attenuate the motor impairment produced by reserpine. Among the tested formulations, CSMNPs (50 mg•kg⁻¹) showed the most pronounced improvement, indicating that incorporation of CS may have enhanced the overall activity of the Se NP formulation.

There is a decrease in motor coordination in rats with Parkinsonism. An increase in the time of fall in the rotarod indicates improved motor coordination and neuroprotection [82]. A decrease in the cataleptic score predicts the antiparkinsonian effect. MP seed*s* are reported to possess anti-cataleptic activity [75]. The results of the present study revealed that reserpine (0.2 mg•kg⁻¹) treated rats exhibited a significant (*p* < 0.05) increase in cataleptic behaviour, indicating severe motor impairment. Administration of L-DOPA, MP extract, MSNPs, and CSMNPs significantly (*p* < 0.05) decreased the cataleptic response as compared to the disease control group, while CSMNPs (50 mg•kg⁻¹) treated rats demonstrated the most pronounced reduction in cataleptic score among all other treatment groups. The enhanced effects of NPs may be attributed to improved bioavailability, sustained release, and enhanced antiparkinsonian activity. The statistical significance of the results was further supported by Cohen’s d analysis, which establishes the significance in relation to the group size and its impact on the treatment.

Furthermore, to confirm the neuroprotective effects of MP extract, MSNPs, and CSMNPs, antioxidant assays were performed using the in vivo model. The levels of antioxidants, including SOD, GSH, and catalase, were evaluated in a reserpine-induced PD rat model. SOD reduces damage at the cellular level by attenuating the production of ROS. It serves as a defence mechanism for oxidative stress and detoxifies superoxide free radicals [64]. The study revealed that the SOD content gradually decreased after reserpine administration. Restoration of SOD following treatment with MP extract, MSNPs, and CSMNPs suggests that these formulations may help strengthen the antioxidant defence against reserpine-associated oxidative stress. Compared to other animal groups, the administration of CSMNPs (50 mg•kg^− 1^) yielded more beneficial effects. Glutathione peroxidase acts on the substrate reduced glutathione (GSH), which generates the free radical that acts as a defence mechanism. However, the GSH content is generally low in the brains of the rats that were induced with PD using reserpine. The MP extract (50 mg•kg^− 1^, p.o.), MSNPs (25 and 50 mg•kg^− 1^), and CSMNPs (25 and 50 mg•kg^− 1^, p.o.) significantly (*p* < 0.05) elevated the GSH levels. The CSMNPs (50 mg•kg^− 1^, p.o.) yielded better outcomes than the other animal groups.

In the rat brain, the NPs control the enzymatic activity of several antioxidants [76]. Catalase is the essential enzyme for converting H_2_O_2_ into water and oxygen, which reportedly detoxifies the generated SOD [77]. The catalase content decreases after the administration of reserpine, which contributes to the development of PD. The present study revealed that treatment with MP extract, MSNPs, and CSMNPs significantly reversed the effect of reserpine-induced PD due to the induced oxidative stress; there was a notable decrease in NO levels in the current PD model. In lipid peroxidation, the MDA level increased, indicating the overproduction of malondialdehyde (MDA) in the brains of reserpine-treated animals. The higher the MDA levels, the greater the likelihood of weakening the antioxidant activity of enzymes like SOD. Our research showed that MP extract, MSNPs, and CSMNPs gradually decrease MDA production. So, we can say that the CSMNPs (50 mg•kg^− 1^, p.o.) have a more protective effect than the MP extract against oxidative stress [78].

The rats treated with reserpine had a significantly (*p* < 0.05) greater nitrite level than the control group. However, the impact was noticeably reversed in the treatment group that received MP extract, MSNPs, and CSMNPs. In rats, a decrease in the brain’s nitrite content indicates a decrease in nitrosative stress and an improvement in antiparkinsonian behaviour.

Histopathological evaluation of the hippocampal region and the substantia nigra of rat brains (*n* = 3) revealed neurodegenerative alterations, including neuronal loss, necrosis, pyknosis, and shrinkage of dopaminergic neurons in reserpine-induced PD rats. Accumulation of Lewy body-like inclusions was also observed in PD rats. Our present study revealed that treatment with L-DOPA, MP extract, MPNPs, and CSMNPs reduced the pathological alterations and preserved normal neuronal architecture in reserpine-induced PD rats. MSNPs and CSMNPs increased the potency and efficacy of MP aqueous extract against reserpine-induced PD.

Collectively, the findings of the present study suggest that MSNPs and CSMNPs possess promising antiparkinsonian and neuroprotective potential in reserpine-induced PD rats. The behavioural, biochemical, and histopathological findings together indicate that attenuation of oxidative and nitrosative stress may contribute to the observed protective effects. Among the tested formulations, CSMNPs, at 50 mg•kg⁻¹, demonstrated comparatively greater overall efficacy. This enhanced response may be related to the combined effects of Se, the bioactive constituents of *M. pruriens*, and the stabilising properties of chitosan. Nevertheless, further studies are required to establish the precise molecular mechanisms underlying these effects and to confirm the contribution of individual components of the formulation.

## Conclusion

The study involved the synthesis and characterization of MP seed extract, MSNPs, and CSMNPs. Both in vitro and in vivo studies revealed the antiparkinsonian properties of MP extract (50 mg•kg^− 1^, p.o.), MSNPs (25 and 50 mg•kg^− 1^, p.o.) and CSMNPs (25 and 50 mg•kg^− 1^, p.o.) on reserpine (0.2 mg•kg^− 1^, i.p.) induced PD in the rat model. The present study’s findings showcased the significant reduction in the effect of reserpine- induced PD by improving motor function, grip strength, and antioxidant levels in the brain homogenate. CSMNPs at (50 mg•kg^− 1^, i.p.) exhibited better antiparkinsonian and antioxidant effects as compared to MP extract and MSNPs, as evidenced by improvements in behavioural, biochemical, and histopathological parameters. The enhanced efficacy and potency of the polymer-tailored metal nanocomposites from MP extract exhibited a remarkable antiparkinsonian effect compared to that of crude MP seed extract and the metal NPs alone.

## Limitations of the study

Although CSMNPs demonstrated improvements in behavioural, biochemical, and histopathological parameters, the study has its own drawbacks. Direct evidence of enhanced BBB penetration and brain delivery was not established. Studies involving biodistribution, brain uptake, pharmacokinetic profiling, and quantitative determination of selenium or L-DOPA levels in brain and other tissues warrant further investigations. The relatively small sample size used for the biochemical and histopathological assessments (*n* = 3 per group) may also have limited the statistical power and robustness of these findings. Furthermore, the reserpine-induced model primarily reflects dopamine depletion and motor dysfunction and does not reproduce the full spectrum of pathological features associated with PD. Therefore, confirmation in complementary PD models is needed to further support the neuroprotective effects observed in this study. In addition, the histopathological findings were based on qualitative observations without quantitative neuronal counting, blinded scoring, or image-based analysis. Further studies incorporating pharmacokinetic and biodistribution assessments, tissue-level quantification, and quantitative histopathological analysis are required to clarify the brain delivery and mechanistic basis of the observed effects.

## Data Availability

The datasets generated during and/or analysed during the current study are available from the corresponding author on reasonable request.
